# Balancing choice and socioeconomic realities: analyzing behavioral and economic factors in social oocyte cryopreservation decisions

**DOI:** 10.3389/fendo.2024.1467213

**Published:** 2024-12-20

**Authors:** Limor Dina Gonen

**Affiliations:** Department of Economics and Business Administration, Ariel University, Ariel, Israel

**Keywords:** cryopreservation, theory of planned behavior (TPB), conjoint analysis (CA), health economics, social oocyte cryopreservation

## Abstract

**Purpose:**

This research investigates the influence of personal income, the likelihood of pregnancy from cryopreserved oocytes, and the risk of infertility, on the decision-making process of women. The study employs the economic stated preference framework alongside the Theory of Planned Behavior in order to comprehend the process of decision-making.

**Design/methodology/approach:**

The data had been collected from women between the ages of 18 and 65 via questionnaire employing conjoint analysis (CA). Through the utilization of this methodology, the factors influencing women’s choices concerning oocyte cryopreservation were quantified.

**Findings:**

The study identified crucial factors that impact the determination to cryopreserve oocytes, such as personal financial resources, the likelihood of achieving a successful pregnancy using frozen oocytes, and the potential for infertility. The analysis reveals that a considerable number of participants perceive cryopreservation as a feasible alternative for augmenting their prospects for future procreation.

**Research implications:**

The results validate the patterns and the ways in which personal and socioeconomic elements impact choices regarding fertility. This has the potential to inform forthcoming health policies and educational initiatives that aim to provide more comprehensive support for women’s fertility decisions.

**Social implications:**

The research highlights the necessity of policy and societal support for women who are contemplating oocyte cryopreservation. It is recommended that public health policies incorporate provisions for state financing of cryopreservation in order to safeguard reproductive autonomy and alleviate the fertility risk linked to the aging process.

**Originality/value:**

His research is unique in that it employs the Theory of Planned Behavior and an economic stated-preference framework to analyze the dynamics of oocyte cryopreservation decisions. This work enhances the existing body of literature by drawing attention to the socio-economic persona factors that influence choices regarding fertility preservation.

## 1 Introduction

Oocyte cryopreservation, or egg freezing, has emerged as a vital reproductive technology, enabling women to preserve their fertility for personal, socio-economic, or health-related reasons. Initially developed for patients facing fertility-compromising medical interventions, oocyte cryopreservation has lately become popular among healthy women seeking to postpone childbirth for career, personal ambitions, or the lack of a suitable partner. This transition not only indicates broader cultural trends in delayed parenthood but also brings about societal benefits, emphasizing the necessity of access to fertility preservation for women.

This study examines the decision-making process influencing women’s decisions to engage in oocyte cryopreservation, including the Theory of Planned Behavior (TPB) alongside an economic stated-preference framework. This research aims to quantify how women navigate financial restrictions, success probability, and perceptions of infertility concerns. The findings of this study offer essential insights for policy formulation aimed at promoting equal access to fertility preservation technologies, underlining the urgency of addressing the economic and social factors influencing reproductive decision-making.

## 2 Contextual review of fertility preservation literature

### 2.1 Introduction to oocyte cryopreservation

Oocyte cryopreservation, also known as egg freezing, represents a significant advancement in reproductive technology, initially developed to preserve the fertility of women undergoing treatments such as chemotherapy, which could jeopardize their reproductive potential (1).

### 2.2 Technological advances in oocyte cryopreservation

The advent of vitrification (“fast freezing”) and intracytoplasmic sperm injection (ICSI) techniques reignited interest in egg freezing in the early to mid-2000s (2). By 2012, authoritative bodies such as the American Society of Reproductive Medicine (3, 4) and the European Society of Human Reproduction and Embryology (5) had removed the experimental status of the procedure, recognizing its safety and efficacy. This paved the way for its broader acceptance and eventual endorsement for non-medical, or ‘social,’ egg freezing, adopted by healthy women who wish to delay childbearing for socio-economic reasons or due to the lack of a suitable partner (1, 3–8).

### 2.3 Medical vs. social egg freezing

Oocyte cryopreservation, also known as egg freezing, has two primary objectives: Medical Egg Freezing (MEF), which preserves fertility for medical causes such as chemotherapy, and Social Egg Freezing (SEF), typically employed for delaying parenthood owing to professional ambitions or the lack of a suitable spouse (3, 7, 9).

The distinctions between medical and social egg freezing significantly affect its accessibility, impacting debates on whether social egg freezing should be regarded as preventative healthcare or an elective choice. These disparities influence access, funding, and broader ethical discussions around reproductive rights, autonomy, and healthcare obligations.

The complex terminology associated with egg storage reflects the ongoing debate regarding whether a traditionally non–medical procedure has a legitimate medical justification. A key question in the field of egg freezing is how to distinguish between “medical egg freezing” (MEF) and “social egg freezing” (SEF) (10).

The notion of medical necessity is a complex issue that requires diverse perspectives for a comprehensive understanding. It is subject to varying interpretations by patients, healthcare providers, politicians, and ethicists. The debate centers on whether age–related fertility reductions should be considered medical conditions. Critics argue that egg freezing is a form of preventative medicine rather than necessary medical therapy (11). This discourse, which addresses both MEF and SEF within a unified framework, informs policy discussions that aim to foster reproductive autonomy across diverse socio–economic circumstances (12–14).

### 2.4 Social egg freezing: managing reproductive options within socio–economic and biological limitations

In the past two decades, oocyte cryopreservation, particularly SEF, has become increasingly recognized as a choice for women wishing to postpone childbirth for non–medical purposes. Research suggests that SEF frequently encompasses job aspirations, financial limitations, and the lack of a suitable partner, underscoring SEF as a reaction to intricate socio–economic and psychological pressures rather than physical conditions (3–5). This discretionary selection also encompasses broader issues such as reproductive autonomy, and the right of individuals to make their own decisions about their reproductive health. SEF not only ignites discussions on the medicalization of societal problems and the ethical ramifications associated with its use, but also empowers women to take control of their reproductive health, making their own decisions about their genetic heritage, familial relationships, and future parenting (9, 15–20).

SEF enables women to preserve fertility while considering the natural decline in reproductive potential linked to age. Research indicates that women, especially those who are highly educated, gainfully employed, and generally aged 36 to 40, engage in SEF as a proactive strategy to counteract age–related reproductive deterioration (12–14). Prevalent factors for deferring motherhood encompass pursuing career stability, attaining educational objectives, financial security, and searching for a suitable spouse (12, 13, 21). Although SEF adopts a proactive strategy for fertility management, the long–term outcomes remain ambiguous, and apprehensions over the effectiveness of reproductive therapies as women age continue to exist (12, 22, 23).

Biologically, the female reproductive window is considerably more limited than that of males, with a pronounced decrease in fertility potential after age 35. The decline is primarily attributable to the diminished quantity and quality of oocytes, which decreases the probability of successful fertilization and heightens the risks of abnormal embryos and fetal loss (12, 13). SEF provides women the opportunity to mitigate biological limitations, therefore aligning reproductive decisions with their personal, professional, and socio–economic aspirations for future parenting. This validation of their choices through SEF provides women with a sense of control over their reproductive health, acknowledging and respecting their individual circumstances and decisions.

The increasing interest in SEF highlights the intricate interaction of social, economic, and biological variables influencing contemporary reproductive choices. SEF is a substantial and complex alternative in modern family planning, inciting continuous discourse over reproductive rights, social norms, and the ethical and medical ramifications of managing non–medical issues via fertility preservation (2–4, 12, 13, 19–22). Given these factors, SEF is an appealing resolution for women facing age–related infertility challenges, which are intensified by the trend of delayed motherhood. Epidemiological studies indicate that as fertility declines in women’s mid–thirties due to diminished oocyte quality and quantity, the incidence of involuntary childlessness rises, with many women over the age of 45 turning to donor eggs for conception (24, 25).

### 2.5 Medical risks and health consequences of social egg freezing for mother and child

The medical ramifications for both the mother and the potential child are crucial factors in SEF. The egg cryopreservation process has two main phases: ovarian stimulation and retrieval, and retrieval, followed by cryopreservation. Initially, the oocytes are harvested after ovarian stimulation, then cryopreserved for long–term storage.

The process itself is not without risks: ovarian hyperstimulation, oocyte retrieval, and pregnancy carry specific medical concerns. For instance, women undergoing oocyte retrieval are at risk of Ovarian Hyperstimulation Syndrome (OHSS), particularly when stimulated for egg retrieval (26). Moreover, if a woman opts to conceive later in life, this may pose considerable risks associated with age–related health issues. Women over 45 undergoing *In Vitro* Fertilization with Intracytoplasmic Sperm Injection (IVF–ICSI) treatments may encounter problems due to pre–existing chronic health conditions, thereby heightening obstetric risks and adversely affecting delivery outcomes (27). Concerns arise over the ethics of providing fertility–preserving technologies to healthy, fertile women, which may foster erroneous expectations about success rates and medical feasibility in later life stages (28).

The future child’s health is crucial in assessing the risks linked to SEF. Advanced maternal age is associated with increased rates of problems, including premature deliveries and low birth weights, which are more common in the offspring of mothers aged over 40. Research demonstrates that advanced maternal age correlates with an increased likelihood of adverse newborn outcomes, such as preterm delivery and lower birth weights, which may influence long–term child health (29). The dual influence on maternal and child health underscores the need for meticulous evaluation from both medical and ethical viewpoints when assessing the function of elective egg freezing in fertility preservation and delayed parenthood.

### 2.6 Critiques, societal implications, and ethical disputes contextualizing social egg freezing

Initially conceived as a medical procedure, egg freezing has become more popular among healthy women desiring reproductive autonomy, raising questions over its categorization, ethical considerations, and matters of public funding (15, 17, 30). SEF is frequently perceived as addressing socio–economic pressures rather than strictly medical issues. Critics argue that offering a medical solution for social challenges—such as career demands and gendered labor market expectations—reflects a “medicalization” of social problems, where individual medical procedures are applied to fundamentally non–medical issues (3, 5, 31–34). Critics propose alternate strategies, including supportive family policies, cultural transformations, and public health initiatives, that could more effectively tackle the structural factors contributing to delayed childbearing (34).

The binary classification of egg freezing into ‘medical’ and ‘social’ categories has oversimplified the complex motivations for the procedure. This oversimplification raises questions about its suitability for regulatory and funding purposes. Van de Wiel (35) argues that this classification fails to fully capture the diverse reasons behind women’s choice of SEF, while Pennings (16) suggests that the ‘social’ label implies that SEF is seen as a preference rather than a necessity. The merging of medical and elective procedures complicates the ethical landscape, making the distinction between these categories problematic, if not impractical. However, the distinction between MEF and SEF continues to have a significant impact on regulatory policies and funding decisions worldwide (15, 17, 30, 35). It is crucial that we develop a more nuanced understanding of SEF to address the ethical complexities in reproductive health.

Advocates of SEF view it as a tool that can significantly enhance women’s autonomy, giving them control over their reproductive timing and helping them overcome the biological constraints that often put them at a disadvantage compared to men in terms of fertility (9, 10, 20). This perspective supports SEF as a legitimate form of reproductive control, arguing that it strengthens women’s autonomy against cultural pressures that may restrict their reproductive choices. The discussion around SEF’s classification underscores the ethical complexities associated with its use and influences policy debates and the availability of this reproductive technique in various socioeconomic contexts (3, 16, 31, 32, 35). The impact of SEF on women’s autonomy is a significant step towards promoting gender equality in reproductive health.

### 2.7 Ethical implications and counselling considerations in elective oocyte cryopreservation

The rapid advancement of assisted reproductive technology (ART) has sparked considerable ethical arguments, a topic thoroughly examined in the literature (36).

The American Society of Reproductive Medicine’s Ethics Committee has deemed planned oocyte cryopreservation ethically acceptable, highlighting its advantages for social equity and women’s reproductive autonomy (37). The first successful birth in the U.S. using vitrified human oocytes was reported in 2013 (38). In France, oocyte vitrification was legalized under the French Bioethics Law of 2011, though it continues to be a subject of debate (27, 39, 40). Meanwhile, the National Bioethics Council in Israel has recommended oocyte cryopreservation to counteract age–related fertility declines (41), whereas in EU countries like Austria, egg freezing for social reasons is currently prohibited but remains a controversial issue (42). While the practice of freezing oocytes for cancer patients and others with decreased fertility is generally viewed positively from both medical and ethical perspectives, extending this option to healthy women for the reasons stated above introduces new ethical debates (36, 43). SEF generates a multitude of ethical considerations, including commercial exploitation, the medicalization of reproduction, the autonomy of women, idealized conceptions of the ideal time to become pregnant, the repercussions of egg freezing on gender disparities, and adherence to professional standards (13, 44, 45). Ethical considerations encompass a comprehensive evaluation of the advantages, disadvantages, costs, and ramifications necessary to guarantee the continued efficacy and safety of the procedure (13, 45).

The benefits that elective egg storage provides to women and its contribution to gender equality are emphasized by proponents of the practice. Many women perceive egg freezing as a method to temporarily suspend their biological cycles, thus safeguarding them against age–related fertility concerns and granting them reproductive autonomy and the potential to conceive biological offspring. Additionally, by freezing oocytes at an earlier stage, the probability of genetic abnormalities developing in offspring may be reduced, this risk increases as the age of the mother increases (44, 46).

Conversely, ethical objections to fertility preservation for non–medical purposes highlight the potential for cryopreserved oocytes to provide illusory optimism for future conception, thereby prompting women to postpone motherhood. A delay may elevate hazards related to late pregnancy for both the mother and child, along with potential repercussions for the child’s psychosocial development stemming from the parent’s older age. Additionally, the fact that many women who opt for SEF ultimately do not utilize their stored oocytes serves as a further critique of the practice (13, 45).

An important counseling point often raised by patients concerns the optimal timing for using SEF. Historically, the typical age for egg freezing or vitrification has ranged from 35 to 38 years (37, 47). Generally, there are two prevailing philosophies regarding SEF. On one hand, experts recommend not delaying the procedure, as older oocytes are less likely to lead to a successful pregnancy due to age–related decline and an increased likelihood of chromosomal aneuploidy. On the other, there is a caveat against utilizing this method at a young age when there is still a possibility that the patient may ultimately not need to use the preserved oocytes at all.

### 2.8 Family planning and oocyte cryopreservation

There has been a significant increase in the quantity of fertility centers worldwide since 2012 that provide elective oocyte cryopreservation (14). Concurrently, a growing proportion of women are choosing to delay the onset of reproduction due to societal considerations.

The dynamics surrounding family planning have undergone substantial transformations in tandem with the shifting roles of women in recent decades. There has been a significant rise in the average age at which women give birth to their first child worldwide (46, 48). Higher education, professional aspirations, financial implications, and changes in social norms and interpersonal connections have all contributed (49, 50). Conversely, postponing parenthood may have an adverse impact on the reproductive capacity of women, a consequence that is frequently unavoidable rather than elective. Involuntary childlessness can be psychologically stressful (51). Female reproductive potential inevitably and irreversibly declines after the age of 37, with oocyte quantity decreasing by a factor of two exponentials (52). Furthermore, it has been observed that the integrity of chromosomes and the quality of ova produced decline in significance beyond the age of 35. Advanced maternal age is a significant risk factor for early miscarriage, with the risk increasing to 51% for ages 40–44 and peaking at 93% after age 45 (53). The success rates for *in vitro* fertilization (IVF) are around 30% for women under 35, however, these rates decline significantly after this age, with almost no chance of a live birth using their own eggs for women over 45. It’s important to note that alternative methods like adoption or IVF with donor eggs may not be suitable for many women, especially those seeking a genetic connection to their child. These methods might encounter several challenges, including age–related constraints (54).

### 2.9 Behavioral determinants and predictive factors in oocyte cryopreservation decisions: a theory of planned behavior approach

The Theory of Planned Behaviour (TPB) (55–57) provides a systematic framework for comprehending intentions, which are significant indicators of behavior. Intentions concerning oocyte cryopreservation stem from a confluence of various factors, including personal characteristics, emotions, intellect, values, and in general attitudes, sociodemographic variables such as age, gender, familial status, educational attainment, and income, as well as the powerful influence of society, including culture, political environment, and social norms (58–60). Behavioral aphorisms represent the consequences individuals link to fertility–related choices, whereas normative beliefs relate to the perceived degree of society’s endorsement of reproductive alternatives, particularly cultural expectations on cryopreservation. Control beliefs include perceptions of the circumstances that either promote or obstruct the choice to freeze oocytes.

A critical aspect of TPB is the concept of subjective norms^1^, which refers to an individual’s perception of psychological support or social pressure from their close social circle to either pursue or avoid a specific behaviour, such as oocyte cryopreservation. While subjective norms may not consistently reflect actual societal perspectives, they considerably influence decision–making, with favorable subjective norms enhancing the probability that a person would opt to cryopreserve oocytes (58, 61).

Another crucial component is perceived behavioral control, which refers to an individual’s evaluation of the complexity or ease of executing the behavior. How a woman perceives the cryopreservation process—whether it is within her control and accessible or challenging and costly—affects her intention to proceed, as subjective evaluations of feasibility strongly influence intentions (58, 61).

To gain a precise understanding of attitudes toward cryopreservation, it is necessary to collect data on various influential factors through comprehensive questionnaires that analyze attitudes, beliefs, and preferences. The TPB–aligned questionnaires in this study aim to comprehensively evaluate many factors affecting oocyte cryopreservation intentions. These factors include emotional responses to potential infertility, which frequently reflect values, general attitudes, worries, risk aversion, resource constraints, and social norms. Factors related to life stages, including age, education, income, religion, and family status, are assessed for their influence on decision–making. Factors such as financial expenses and the assessment of utility, which encompasses both private and social advantages, further influence an individual’s intentions about cryopreservation (62).

### 2.10 Cost–effectiveness and informed decision–making in social egg freezing

Elective oocyte cryopreservation is a decision that should not be taken lightly. It requires careful consideration and is typically guided by a multidisciplinary team, including an embryologist, fertility expert, and psychologist or counselor. Their role is to help women make informed decisions by understanding the procedure’s risks, benefits, and associated costs. This involves discussions on success rates, potential long–term health implications, statistics on offspring conceived from cryopreserved oocytes, the duration of egg storage, and the importance of signing an informed consent document (2, 13, 63, 64).

It’s important to note that the use rate of frozen oocytes is relatively low, with studies showing a range of 6% to 15% from a cost–effectiveness perspective (27, 41, 65–68). This underscores the need for careful consideration of the economic aspects of elective oocyte cryopreservation, ensuring that resources are used efficiently. Success rates tend to be higher when women opt for oocyte vitrification at a younger age because fewer vitrified oocytes are required to achieve a live birth. Paradoxically, however, younger women are less likely to use their vitrified oocytes due to a greater probability of finding a partner and conceiving naturally later on. Van Loendersloot et al. (69), Hirshfeld–Cytron et al. (70), Garcia–Velasco (71) Mesen et al., and Devine (72, 73) suggest that oocyte cryopreservation would be cost–effective if at least 50–60% of women actually use their vitrified oocytes. On the other hand, other scholars (74–76) contend that a 50% utilization rate may be excessively optimistic. They suggest that women at a higher risk of becoming prematurely sub–fertile—because of factors like ovarian endometriosis, ovarian surgery in the past, or personal circumstances that prevent them from getting pregnant—are more likely to use their vitrified oocytes. Oocyte freezing is probably more economical for these women.

### 2.11 Social benefit and public funding

The discourse on societal benefits is crucial when evaluating public financing for procedures such as oocyte cryopreservation, presenting the challenge of who ought to bear the costs.

‘Elective freezing,’ ‘non–medical freezing,’ or ‘social freezing’ (as opposed to ‘medical freezing’) is currently a privilege mostly enjoyed by women who can afford the costs of ovarian stimulation medicines, medical procedures, vitrification or slow freezing, and storage fees (77).

While the right to propagate is commonly recognized as a liberty right, it is not typically considered a claim right (78). This means that although women may elect to cryopreserve their oocytes, they do not have a legitimate claim on societal resources to subsidize it. However, many Western countries offer healthcare coverage for a certain number of ‘standard’ IVF cycles to ensure equal access, and several US states mandate infertility insurance coverage. This suggests that, in these jurisdictions, the right to reasonable healthcare extends to fertility treatments (79).

The question arises: should countries with publicly funded IVF extend coverage to social freezing? If oocyte cryopreservation is an accepted method to counter infertility and fertility treatment is covered by public healthcare, should social freezing also be included in public healthcare or mandated insurance coverage, or is there a significant distinction between ‘regular’ IVF and IVF with previously stored oocytes? The challenge in this assessment is that elective oocyte freezing involves two distinct phases: initially, ovarian stimulation, oocyte retrieval, cryopreservation, and storage, and later (often years afterward), the thawing and fertilization of the cryopreserved oocytes. In the first phase, women who opt for social freezing are healthy and are seeking a procedure that results in stored oocytes that may or may not be utilized, depending on their life circumstances. The second phase is medical intervention. Women seeking elective oocyte cryopreservation differ from other IVF patients in a critical way: they are not infertile, which is often a prerequisite for state–funded IVF cycles in many countries.

The term ‘elective freezing’ highlights that oocyte cryopreservation by healthy women is similar to other elective medical interventions, like cosmetic surgery, which typically do not provide direct therapeutic benefits (unless psychologically). This raises questions about why society should fund what some might consider merely a convenience. However, while the distinction between medical and social interventions often guides reimbursement policies, there are many exceptions, particularly in reproductive health, such as elective abortion, contraception, and pregnancy care, which are treated as medical interventions deserving of coverage despite pregnancy not being a disease (80).

Furthermore, social freezing may be conceptualized as a type of anticipatory medicine in which women reserve eggs in anticipation of potential future reproductive difficulties. While this preventative strategy may not yield immediate therapeutic advantages, it does possess the potential for future therapeutic benefits, which may support its inclusion in healthcare coverage. If public healthcare covers IVF cycles using fresh but aged oocytes or donor oocytes for women, it follows that the use of their cryopreserved oocytes should also be reimbursed. Consistency in treatment would acknowledge the ethical and practical advantages of using freshly aged oocytes from the woman herself instead of utilizing donor oocytes. These considerations support the idea that compensation for elective cryopreservation should be comparable to that for “regular” IVF treatment when viewed as a unified entity with IVF treatment. However, a more nuanced policy approach may be needed given the separate steps of the process and the possible absence of causality between the initial storage phase and the subsequent treatment phase. Options might be full coverage or a cash or service refund for the first phase in the event that the woman returns for the second.

### 2.12 International regulatory landscape

Research conducted by the European IVF–monitoring Consortium (EIM) for the European Society of Human Reproduction and Embryology (ESHRE) and its Working Group on Oocyte Cryopreservation highlights considerable variability in the regulatory and financial frameworks governing egg freezing across forty–three countries. The data indicates that legal frameworks and financial mechanisms vary significantly, illustrating diverse national strategies for managing and promoting this technology (81, 82).

Over the past thirty years, there has been a noticeable increase in the postponement of motherhood among women of reproductive age in several Western nations. This trend is primarily attributed to a variety of factors, including improved educational and professional opportunities, caregiving responsibilities, financial challenges, the pursuit of economic security, the absence of a suitable partner, and the aspiration to establish a stable home environment. Additionally, the widespread availability of contraception and the belief that individuals are not yet ‘ready’ for motherhood further contribute to this shift (83).

When it comes to the funding of medical and SEF, policies vary significantly. For instance, nations like Israel, the United States, and certain European regions provide either partial or full coverage for MEF. However, SEF is often excluded from public funding due to its optional nature. In Israel, public funding for MEF is allowed through the national health insurance system, but SEF is limited to private healthcare plans. This distinction underscores the emphasis on medically necessary applications over optional procedures (11, 34, 82).

### 2.13 Israel

Under the Israeli National Health Insurance Law (1994), which ensures the accessibility and financial support of numerous technologies, including IVF, the funding and utilization of Assisted Reproductive Technologies (ART) are regulated in Israel. Egg donation is an example of an ART that is regulated by specific laws (10), as opposed to directives from the Israeli Ministry of Health regarding other ARTs. Two directives specifically dedicated to regulating oocyte vitrification were issued by the Israeli Ministry of Health. The first directive (84) stated that vitrification should no longer be considered experimental. The subsequent directive (85) detailed the indications and conditions that justify the use of egg freezing, allowing both MEF and SEF.

The Israeli National Health Insurance Law (1994, section 6 amended in 2011) outlines chemotherapy and radiation therapy as justifiable indications for funding fertility preservation methods such as egg–freezing, embryo freezing, and ovarian tissue freezing (children, adolescent, and women patients). The Israeli Ministry of Health (86) also provides similar indications based on recommendations from the Israeli National Council for Gynecology, Neonatology, and Genetics. In 2011, the Ministry published additional medical conditions under which egg freezing will be performed, extending indications for medical fertility preservation beyond cancer patients to include other conditions and procedures that pose a risk to future fertility.

While MEF and SEF are regulated and performed in Israel, the funding guidelines differ. Fertility preservation is fully covered by the Israeli National Health Insurance for medical indications (87). Women undergoing chemotherapy or radiation do not incur costs for fertility preservation for up to two children (85). For increased risk of early amenorrhea, funding for MEF is limited to women under the age of thirty–nine and to a maximum of four treatment cycles or twenty retrieved eggs—whichever comes first. If the woman is a carrier of Fragile X Syndrome^2^, funding extends to six cycles or forty eggs (88). Funded storage is limited until the birth of two children or until the woman reaches the age of forty–two (whichever is earlier).

In contrast, SEF is not covered by the Israeli National Health Basket. However, one Health Maintenance Organization, “Meuhedet,” offers partial subsidization for women with supplemental medical insurance (89). The usage of frozen eggs later can be funded as part of the public funding scheme for IVF, with every woman aged eighteen to forty–five entitled to almost unlimited funded treatment up to the birth of two living children, without conditions based on familial status or sexual orientation.

In 2014, some moderate restrictions were issued concerning the provision of IVF, such as reassessment after eight unsuccessful cycles (90). SEF regulations allow healthy women aged thirty to forty–one to freeze eggs, limiting the procedure to four treatment cycles or twenty retrieved eggs (whichever comes first) with implantation of fertilized eggs allowed until the age of fifty–four. Eggs can be stored for five years with an option to extend.

These differences between MEF and SEF can be seen as establishing a hierarchy, prioritizing MEF over SEF in terms of funding and regulatory support.

Regarding Jewish religious tradition and practice, egg freezing has been embraced by Israel’s religious establishment, spanning various local religious factions. The PUAH Institute, established in 1980 to align ART implementation with Jewish law (halacha) (91, 92) has strongly supported egg freezing, particularly advocating for SEF among single Orthodox women and providing support in IVF clinics. PUAH’s official stance on egg freezing suggests that SEF can aid women who began childbearing later in life but wish to establish a family (93) (PUAH Institute 2018). The Institute also extends its support to Jewish American communities, offering educational, financial, and emotional assistance for those utilizing ARTs. Consequently, rabbis across various religious communities now encourage single Orthodox women in their late thirties to freeze their eggs (94).

In Judaism, where reproduction is a central tenet, innovative reproductive technologies that facilitate the growth of the Jewish population are widely accepted (95–97). Jewish women who opt for egg freezing are often seen as committing to the Jewish maternal imperative—the religious and social expectation for Jewish women to engage in “reproducing Jews” (94), “embodying (Jewish) culture” (98), and “birthing a mother” (99). This imperative is particularly prominent in Israel, described as the “land of imperative motherhood” [98[, where the state supports Jewish women’s reproduction through numerous subsidized fertility services. Israeli women and couples may even undergo various forms of “bio–scrutiny^3^” to ensure they create the desired type of Jewish family in terms of both physical and genealogical heritage (100).

#### 2.13.1 The Jewish maternal imperative

Childbearing holds a revered place in Judaism, where both ancient and modern texts view it as essential to personal and social identity and vital for the continuity of the Jewish people, giving it a collective moral significance (95, 101). While the commandment to “be fruitful and multiply” traditionally applies to men, Jewish identity is passed matrilineally, making childbearing a significant responsibility and life goal for Jewish women. Most rabbis assert that an infant’s Jewishness depends on the mother’s religion—emphasizing the womb over genetic lineage.

However, recent anthropological studies highlight the importance of genetics in contemporary Jewish reproduction, underscoring the preference for using one’s own eggs (95, 98, 99, 102–108). In Israel, childbearing is not only critical for nation–building (106, 107) but also considered a primary form of women’s political participation (109–114), resulting in Jewish women in Israel having more children on average than those in any other industrialized nation. Childlessness carries a significant stigma, often overshadowing other life achievements (99).

Viewing “the right to parenthood” as a fundamental human right (95), childless women in Israel often describe their condition as akin to a “serious illness,” with infertility perceived as a “final extinction” for families of Holocaust survivors (95). The enthusiastic reception of all forms of ARTs since the early introduction of IVF (95, 97, 115, 116) supported by the world’s most generous state–backed IVF policy (117), illustrates the deep commitment to the Jewish maternal imperative. Despite the intense physical and emotional toll of these procedures (118), many women persist with these invasive treatments, viewing them as pathways to fulfillment and happiness (119).

### 2.14 Future research directions

Further research is necessary to deepen the understanding of the long–term societal, health, and familial impacts of oocyte cryopreservation. Emerging technologies, demographic shifts, and evolving societal attitudes towards delayed parenthood present significant areas for study. Longitudinal research on the health outcomes of children born from cryopreserved oocytes will provide valuable insights into potential long–term effects. Additionally, studies on the psychological and social impacts of egg freezing, particularly for women who eventually do not use their stored oocytes, will contribute to a holistic understanding of this practice. Investigation into cost–effectiveness, alongside policy and regulatory impacts across various regions, will also aid in formulating equitable, accessible, and sustainable fertility preservation strategies.

### 2.15 Conclusion

Oocyte cryopreservation is a transformative option in reproductive healthcare, empowering women with increased control over family planning and allowing them to navigate the intersection of career, personal goals, and biological limitations. As healthcare policies and societal norms continue to evolve, it is crucial to balance access, ethical considerations, and cultural influences to support reproductive autonomy. This study underscores the importance of establishing policies responsive to the multifaceted needs of women, contributing to a framework that respects both individual choices and broader societal impacts.

## 3 Methods

### 3.1 The theoretical framework

Through the integration of the economic stated preference framework and the TPB, this study seeks to investigate the motivations underlying cryopreservation. The TPB (55–57) is employed to underscore the correlation between micro and macro–level intentions and behaviors. Fertility behavior is perceived as the result of a decision–making process that weighs the advantages and disadvantages of potential courses of action within the micro context with consideration for individual characteristics and variables. These factors include subjective norms (an individual’s perception of psychological support or social pressure) and perceived behavioral control (how easy or difficult an individual perceives it is to perform the behavior or reach the intended goal), which both significantly influence intentions and behaviors (62). The approach uses questionnaires to assess elements such as beliefs, attitudes, and social norms (for example, norms concerning the appropriate age for childbearing), as well as wider national or cultural values, and the economic and political environment. Furthermore, the TPB is evaluated at the macroeconomic level (59, 120), specifically regarding how governmental entities determine whether to subsidize or finance oocyte cryopreservation.

### 3.2 The empirical model

Insights into the preferences and evaluations of patients regarding various facets of healthcare procedures are critical for program development and assessment. By incorporating patient preferences into policy decisions concerning clinical practices, licensing, and reimbursement, substantial improvements can be achieved. Enhancing the alignment of healthcare policies with patient preferences has the capacity to elevate satisfaction levels with clinical interventions and public health undertakings, thereby potentially bolstering the overall efficacy of healthcare processes (121, 122).

Economists define two main approaches to measuring preferences: revealed and stated (123). Revealed preferences are inferred from actual observed behaviors in the market, identified through complex econometric methods used by researchers. In contrast, stated preferences are gathered through surveys that allow researchers to manipulate how preferences are elicited.

Stated–preference methods are categorized into two main types: Methods that utilize rating, ranking, or choice designs (used individually or in combination) to quantify preferences for various attributes of an intervention. These methods, commonly referred to as conjoint analysis (CA), discrete–choice experiments, or stated–choice methods, are designed to explore the trade–offs between different properties of a product and their influence on user preference (124–126).

### 3.3 Conjoint analysis in health care studies

The use of CA in healthcare research has increased substantially (127, 128), with Clark et al. (129) and De Bekker–Grob et al. (130) providing exhaustive literature reviews. CA is a method that, based on the evaluation of a set of values (131–134), derives part–worth values for individual attributes from a total score for a product or service composed of multiple attributes. This methodology is especially well–suited for quantifying preferences for non–traditional market products and services or those in sectors where market options are limited by regulations or legal restrictions, such as healthcare (135). CA has demonstrated efficacy in preference measurement across a multitude of health applications (89, 128, 136–141), and its applicability transcends healthcare interventions. It is increasingly used to understand preferences related to health–related quality of life and to evaluate patient–reported outcomes of different health conditions (142, 143). Licensing authorities have also shown interest in CA as a tool for assessing patient willingness to undergo innovative treatments that may offer enhanced efficacy (144).

CA facilitates decision–making processes for patient participation (145, 146), supports shared decision–making (147), aids in clinical decision–making (148), and helps elucidate how various stakeholders value healthcare outcomes (149). Furthermore, CA can evaluate the relative importance of one or more attributes of a product or service and assess how individuals make trade–offs between these attributes. This process identifies the user–required exchange rate between units of an attribute (149).

CA studies present hypothetical scenarios to participants, which involve attributes of a product or service that are assigned to different degrees of importance. Respondents are then asked to rank these services, rate them, or choose between paired attributes. While people frequently make decisions involving exchange and substitution in their daily lives, they are rarely required to explicitly rank and rate attributes as part of their routine decision–making. This paper contributes to the development and application of the pairwise ‘choice’ approach in the decision–making process, which compares two indirect utility (benefit or satisfaction) functions. Participants in the study are asked to make a series of pairwise choices, selecting the option that offers the higher level of utility in each comparison.

### 3.4 Study design and methods

CA techniques used to elicit preferences helped determine the relative importance that individuals attribute to different attributes of a particular health product or service (135). By analyzing how participants express their inclinations towards various attributes of the product or service, CA enables the evaluation of the practicality, or implicit worth, of those particular elements of the healthcare intervention. The analysis of CA in this paper is grounded in the methodology described by Ryan (150).

For this research, a structured CA questionnaire was developed: Participants were presented with hypothetical scenarios with various attributes crucial to cryopreservation and asked to make pairwise choices between two options. The initial set of attributes and their levels were defined based on a literature review. **Appendix 1**, **Table 1**, summarizes the attributes and levels included in the CA study. Each respondent was shown a series of 10 scenarios, with Option A having fixed attributes and Option B varying in each scenario, thus forming a total of 10 pairwise choice questions. An example of one such pairwise choice is detailed in **Table 2** of **Appendix 1**.

### 3.5 Experimental design & methods

#### 3.5.1 Data collection

Data was gathered by surveys conducted among women from the general public. The study respondents were drawn from a pool of participants recruited through a survey company and participated voluntarily without any monetary compensation. The study design was cross–sectional^4^ with a single data collection point. The survey company had sole access to the participants’ data. Each participant was given a personal code so that the personal information was not known to the researcher conducting the study. Participants were given detailed information on oocyte cryopreservation prior to completing the survey. This included explanations about the reasons for considering oocyte cryopreservation, such as medical conditions like cancer, military service risks, and high–risk occupations, as well as the biological background regarding a woman’s egg reserve and the benefits of egg freezing (the Questionnaire is presented in **Appendix 2**).

#### 3.5.2 Questionnaire design

Three data–gathering stages were used to construct the survey and carry it out:

Preliminary Stage: In the preliminary stage, items to be included in the research questionnaires were identified, using in–depth interviews with five fertility experts and three potential candidates for oocyte cryopreservation. The time frame for the preliminary interviews was six months. The questionnaires were initially constructed on the basis of content analysis of interview results.

Pilot Study: After completing the first version of the questionnaires (based on the preliminary stage findings), a pilot study was conducted, with 15 participants. The pilot study aimed at assessing the difficulty and clarity of the questionnaire and the respondents’ willingness to respond to the items in it. This pilot study, which included face–to–face interviews conducted by the researcher, provided the participants with detailed information about cryopreservation and enabled the presentation of relevant information in a supervised manner as it gathered responses to the different factors. The time frame for the pilot study was three months.

Main Survey: Based on the findings of the pilot study, the research questionnaires were developed for the survey population. The population sample consisted of Israeli Jewish women aged 18–65, from four major urban centers: four large cities in four major population regions in Israel: Tel Aviv, Jerusalem, Haifa, and Beer Sheba. First, the survey company made contact by telephone, then questionnaires via Google Docs were sent to respondents who agreed to participate in the study. Every respondent confirmed her participation by digitally signing an informed consent form. The time frame for the main survey was 3 months.

Out of 807 questionnaires distributed, 94 were eliminated because they had invalid or missing data and 148 were eliminated because of inconsistency i.e., according to internal (theoretical) consistency tested through the CA technique (See Section 4.3 Methodological issues addressed). The final sample consisted of 565 participants.

#### 3.5.3 Ethical considerations

The participants, all 18 years of age and over, were given a page describing the goals of the study, guaranteeing anonymity, and explaining the possibility of terminating their participation at any time. Participants were asked to sign an informed consent form before answering the questionnaire. Anonymous, self–administered questionnaires were filled out without interventions by investigators. In the cover letter attached to the questionnaire, the participants were informed that data collection and analysis would be kept fully anonymous, and their personal information would be fully protected, all answers would be kept confidential, processed statistically, and used for scientific research only.

The participants were free to decide whether or not to participate. Each participant provided signed informed consent to participate in the study. Ariel University Ethics Committee approval number: AU–SOC–YB–20141230.

## 4 Data analysis

SAS Vs 9.4 was used for the analysis.

Continuous variables were presented by mean and standard deviation, or median and inter quantile range. Categorical variables were presented by (N%).

In market research, CA is a statistical method utilized to determine how individuals make purchases and what qualities they genuinely value in services and products. In this type of survey, participants are provided with a series of alternatives or products containing distinct qualities at varying degrees. They are subsequently requested to select their preferred option or arrange them in ranked preference. The basic idea is to dissect and analyze the options in order to ascertain which attribute combination has the greatest impact on consumer choice.

The premise of this methodology is that a product’s qualities (e.g., price, quality, brand, features) characterize it, and that the consumer’s assessment of the product is a composite of the assessments of each individual quality or brand attribute. CA can distinguish the relative significance of attributes that influence a consumer’s choice or decision by presenting them with a variety of product configurations comprised of distinct attribute combinations.

A conjoint analysis is performed as follows in **Table 1**:

**Table 1:** Steps in conjoint analysis design and implementation for healthcare decision-making.

Step	Description
Designing the Study	Defining the product attributes and their corresponding levels to be incorporated in the study. This requires a comprehensive understanding of the determinants that consumers deem crucial when comparing products.
Formulating the Survey	By employing the chosen attributes and levels, an assemblage of speculative product offerings or scenarios is produced. The respondents are presented with these in a manner that facilitates comparison between them.
Data Collection	Participants provide rankings or selections of various product offerings, enabling researchers to gather information regarding consumer preferences. Following this, the survey data are subjected to statistical models for the purpose of estimating the part–worth utilities of the attribute levels. By employing this procedure, the degree to which each attribute level influences the consumer’s choice can be quantified.
Analysis Result Interpretation	The findings may illuminate consumer–preferred attributes, indicate the relative importance of particular attribute levels, and potentially forecast market preferences regarding novel product designs.

**Appendix 1** of the research paper contains the details of the CA study. In the **Appendix 1**, **Table 1** presents a comprehensive overview of the attributes and levels that were assessed throughout the investigation. Ten distinct scenarios, each with two alternatives (Option A and Option B), comprised the study. Option A possessed constant attributes, whereas Option B varied across scenarios. This setup resulted in 10 pairwise choices used in the CA questions.

**Table 2** in **Appendix 1** presents an example of one such pairwise choice.

CA is important for several reasons as described in **Table 2**:

**Table 2:** Key benefits and applications of conjoint analysis in evaluating healthcare preferences.

Benefit	Description
Attribute Importance	CA assists in determining which attributes of a product or service consumers value the most, to help prioritize features during product development.
Trade–offs	In the process of decision–making, consumers frequently make trade–offs among various attributes. By comparing and contrasting the variations between scenarios, organizations can obtain insight into how customers weigh the relative importance of various factors.
Value Quantification	CA offers a quantitative assessment of how much importance consumers attribute to individual levels of each attribute. This data is of the utmost importance in formulating pricing strategies and identifying the features that provide the greatest value to the customer.
Market Segmentation	By analyzing scenarios involving a wide variety of respondents, conjoint analysis can discern discrete segments according to attribute preferences. This enables organizations to concentrate their marketing initiatives and customize their products to target distinct market segments.
Simulation and Prediction	The influence of modifications in product attributes on consumer choice can be simulated using conjoint analysis. Utilizing this predictive capability to evaluate the potential success of new products or modifications to existing ones is invaluable.
Competitive Advantage	By understanding consumer inclinations and compromises, organizations are capable of developing products that more effectively fulfill consumer requirements in comparison to their rivals, potentially offering a market advantage.
Optimization	Organizations can optimize their product offerings to maximize profitability and customer satisfaction by identifying the trade–offs consumers are prepared to make in exchange for the most highly valued attributes.

Using CA to examine the distinctions between scenarios yields comprehensive insights into the decision–making processes of consumers, helping businesses align their products and services with consumer preferences and improving market fit.

Scenarios are presented in **Table 3** in **Appendix 1** – according to the distinction between options B and A.

Exploratory factor analysis of the attributes relevant to the decision to cryopreserve oocyte: Risk of infertility, Chances of success of the oocyte cryopreservation process, Chance of initiating a pregnancy from frozen oocyte, Option of oocyte cryopreservation for chosen period of time (Years), Initial registration fee to fertility laboratory and cryopreservation (One–time payment), Annual fee for cryopreservation and must be paid every year (storage) was carried out using Principal Component Analysis, Rotation Method: Varimax with Kaiser Normalization. This analysis yielded two factors:

1. Factor_Risk=Mean (Risk of infertility, Chances of success of the oocyte cryopreservation process, Chance of initiating a pregnancy from frozen oocyte, Option of oocyte cryopreservation for chosen period of time (years).

2. Factor_Price=Mean (Initial registration fee to fertility laboratory and cryopreservation (one–time payment), Annual fee for cryopreservation and must be paid every year (storage)

The regression function estimated is denoted by Equation 1 and Equation 2.


(1) $$\begin{array}{c} \Delta \text{V}=\text{β}0+\text{β}1\;\text{Risk}\;\text{of}\;\text{infertiliti}\\ +\text{β}2\;\text{Chances}\;\text{of}\;\text{success}\;\text{of}\;\text{the}\;\text{oocyte}\;\text{cryopreservation}\;\text{processi}\\ +\text{β}3\;\text{Chance}\;\text{of}\;\text{initiating}\;\text{a}\;\text{pregnancy}\;\text{from}\;\text{frozen}\;\text{oocytei}\\ +\text{β}4\;\text{Option}\;\text{of}\;\text{oocyte}\;\text{cryopreservation}\;\text{for}\;\text{chosen}\;\text{period}\;\text{of}\;\text{timei}\\ +\text{β}5\;\text{Initial}\;\text{registration}\;\text{fee}\;\text{to}\;\text{fertility}\;\text{laboratory}\;\text{and}\;\text{cryopreservationi}\\ +\text{β}6\;\text{Annual}\;\text{fee}\;\text{for}\;\text{cryopreservation}\;\text{and}\;\text{must}\;\text{be}\;\text{paid}\;\text{every}\;\text{yeari}+\text{ϵ} \end{array}$$

CA was estimated in accordance with the function of the form:


(2) $$\begin{array}{c} \Delta \text{V}=\text{β}0+\text{β}1\text{ Agei}+\text{β}2\text{ Educationi}\\ +\text{β}3\text{ Incomei}+\text{β}4\text{ Religious}\;\text{Observance}\;\text{Rei}\\ +\text{β}5\text{ Religious}\;\text{Observance}\;\text{Tri}+\text{β}1\;\text{Risk}\;\text{of}\;\text{infertiliti}\\ +\text{β}6\;\text{Chances}\;\text{of}\;\text{success}\;\text{of}\;\text{the}\;\text{oocyte}\;\text{cryopreservation}\;\text{processi}\\ +\text{β}7\;\text{Chance}\;\text{of}\;\text{initiating}\;\text{a}\;\text{pregnancy}\;\text{from}\;\text{frozen}\;\text{oocytei}\\ +\text{β}8\;\text{Option}\;\text{of}\;\text{oocyte}\;\text{cryopreservation}\;\text{for}\;\text{chosen}\;\text{period}\;\text{of}\;\text{timei}\\ +\text{β}9\;\text{Initial}\;\text{registration}\;\text{fee}\;\text{to}\;\text{fertility}\;\text{laboratory}\;\text{and}\;\text{cryopreservationi}\\ +\text{β}10\;\text{Annual}\;\text{fee}\;\text{for}\;\text{cryopreservation}\;\text{and}\;\text{must}\;\text{be}\;\text{paid}\;\text{every}\;\text{yeari}\\ +\text{β}11\text{ Factor}\;\text{Riski}\\ +\text{β}12\text{ Factor}\;\text{pricii}+\text{β}13\text{ WTPYearlyi}+\text{ϵ} \end{array}$$

CA was estimated in accordance with the function of the form:

ΔV^5^ = α_1_ *risk of Infertility* ^6^ + α_2_ Chances of success of the oocyte cryopreservation process^7^

+ α_3_ Chance of initiating a pregnancy from frozen oocyte^8^

+ α_4_ Option of oocyte cryopreservation for chosen period of time^9^

+ α_5_ Initial registration fee to fertility laboratory and cryopreservation^10^

+ α_6_ Annual fee for cryopreservation and must be paid every year^11^

+ α_7_ *factor Risk* ^12^ + α_8_ *factor price* ^13^ + α_10_ *WTP* ^14^ + α_11_ *trad*

+ α_12_ *religious* + α_13_ *age* + α_14_ *education* ^15^ + *e* ^16^ + *u* ^17^

### 4.1 Methodological issues addressed

When using the CA technique, it is important to include an evaluation of whether individuals appear to understand the technique and relate to it seriously. This study tested for internal (theoretical) consistency and validity (152).

To check internal consistency, the rationality of the choices made was tested, i.e., if one scenario is clearly ‘better’ than another, respondents are expected to choose that scenario. In choice 7, the expectation is that all respondents would prefer the second scenario over the first. The assumption about respondents who answered inconsistently was either they did not understand the questionnaire, or they were not taking it seriously, these responses were omitted from the analysis. The premise of CA is that individuals have continuous preferences so that a deterioration in the level of one attribute is always compensated for by an improvement in another.

The regression analysis results were used to test the internal validity of CA, i.e., the extent to which the independent variables being tested are what led to the predicted results.

Given that higher levels of risk of infertility imply a problem, one would expect that the coefficient of the attribute ‘Importance of the risk of infertility’ would be positive in the regression equation regarding the WTP for cryopreservation. One would expect the coefficient of the Cost attribute to be negative regarding the WTP for cryopreservation. And one would expect that the coefficient of the attribute Personal monthly income would be positive in the regression regarding the WTP for cryopreservation. 

## 5 Results

### 5.1 Statistical descriptive analysis

A statistical descriptive analysis was performed to investigate the social and demographic characteristics of the respondents who participated. **Table 3** summarizes the social and demographic characteristics of the research sample. Respondents’ Age, Education, Personal Income, Degree of Religious Observance, and Marital Status were included as demographic variables in the model.

**Table 3:** Social and demographic characteristics.

Variables	N=565 %
^18^Age	
22–18	15.60
27–23	46.10
32–28	13.83
37–33	7.09
38–42	8.33
43–47	6.91
48–59	1.60
60–65	0.53
^19^Degree of Religious Observance	
^20^Orthodox – Follow the traditional Jewish religion.	28.85
^21^Ultra–Orthodox – Highly conservative	3.36
^22^Secular – Not religiously observant	35.58
^23^Traditional – Observant of some of the religious tradition	32.21
^24^Education	
^25^Elementary School	0.18
^26^Partial High School	1.77
^27^High School full education	19.47
^28^Post–High School	6.19
^29^Partial Academic degree	28.14
^30^Academic degree Full education	44.25
^31^Personal monthly income $	
<$1,226	36.28
$1,226–$2,145	32.92
$2,145–$3,065	21.59
$3,065–$3,984	5.49
$3,984+	3.72
^32^Marital Status	
Married	58.41
Single	39.46
Divorced	1.95
Widow	0.18

The CA investigation encompassed ten unique scenarios, each of which offered two alternatives (Option A and Option B). Option A maintained consistent attributes across all scenarios, whereas Option B exhibited variations across different scenarios. A total of 10 pairwise comparisons were generated by this design, which were then employed in the Conjoint Analysis (CA) to investigate decision–making preferences.

**Table 3** of **Appendix 1** specifies the 12 different scenarios (cases) in terms of the difference in the characteristics vs the baseline scenario (Option A).

**Table 4** of **Appendix 1** lists, for each of the 12 scenarios (cases) the percentage of participants who chose option A and the percentage who chose the alternative.

**Table 5** in **Appendix 1** lists the mean and standard deviation of values of the scenario parameters, for scenarios which were chosen vs scenarios which were not. The values are shown in absolute terms, instead of differences from option A, as this is easier to intuitively understand. The purpose of this table is to judge the relative differences in parameters between scenarios that were chosen and those that were not. It can be seen that Risk of infertility and Initial registration fee negatively affect choice (were lower for chosen), Chances of success, Chance of initiating a pregnancy from frozen and Option of oocyte cryopreservation positively affect choice and Annual fee for cryopreservation has almost no affect. 

**Table 6** in **Appendix 1** provides descriptive data regarding the distinctions in choice parameters between option B and option A, categorized by the option selected.

**Table 7** in **Appendix 1** presents the Principal Component Analysis. In addition to the presentation of the Principal Component Analysis and factor loadings, two binary logistic regressions were used to estimate the change in utility in moving from one scenario to the second scenario.

### 5.2 Statistical significance of the attributes – CA – Logit model

Binary logistic regression is used to assess the effect of variables on a binary outcome. In this case it is used to assess the effect of the difference in scenario variables, between each scenario and the baseline scenario, on the probability of choosing the baseline scenario. The worth of this statistical model can be measured by the C–Index, the closer this index is to “1”, the higher the model’s ability to discriminate between individuals who chose the baseline scenario vs another scenario.

**Table 4** shows the results of a binary logistic regression analysis.

**Table 4:** Logistic regression for option chosen by difference in scenario parameters.

Parameter	DF	Estimate	Standard Error	Wald Chi–Square	Pr > ChiSq	Standardized Estimate
Intercept	1	0.5743	0.0688	69.6227	<.0001	
Diff_Risk of infertility	1	0.00547	0.00110	24.9195	<.0001	0.0893
Diff_Chances of success of the oocyte cryopreservation process	1	–0.0248	0.00138	320.8853	<.0001	–0.3087
Diff_Chance of initiating a pregnancy from frozen oocyte	1	–0.0386	0.00221	306.2231	<.0001	–0.2996
Diff_Option of oocyte cryopreservation for chosen period of time (Years)	1	–0.2138	0.0145	216.4278	<.0001	–0.2779
Diff_Initial registration fee to fertility laboratory and cryopreservation (One–time payment) _100	1	0.0125	0.000733	290.9027	<.0001	0.2988
Diff_Annual fee for cryopreservation and must be paid every year (storage)	1	0.000606	0.000568	1.1395	0.2857	0.0167

Prior to analysis, the difference in first price was divided by 100, in order to produce a sensible odds ratio. This only influences estimates (beta) and odds ratio, it has no effect on significance or standardized estimate. The Standardized Estimate can be used to derive relative importance of the various factors (Odds ratios depend on unit of measure and cannot be compared between parameters). All variables, except difference in yearly price, were significant.

An Odds Ratio above 1 means that a larger difference between the parameters in option B vs. option A leads to a higher probability of choosing option A. An Odds Ratio below 1 means that a larger difference between the parameters in option B vs. option A leads to a lower probability of choosing option A (See **Table 8** in **Appendix 1**).

The model’s discriminatory ability (C–Index) was 72.9% (See **Table 9** in **Appendix 1**).

From **Table 4** p–values we can see that all scenario parameters, except the difference in annual fee, had a significant effect on the probability of choosing the baseline scenario. From the standardized estimates, we can see those differences in chance of success, cryopreservation process, initiating a pregnancy from frozen oocyte and initial registration fee to fertility laboratory and cryopreservation had similar effect sizes (ranging from 0.28 to 0.31, in absolute value), while difference in risk of infertility had a much lower effect (0.09).

**Tables 8**–**10**, located in **Appendix 1** along with detailed explanations, provide an in–depth statistical analysis of the factors influencing decisions around oocyte cryopreservation. **Table 8** in **Appendix 1** highlights the odds ratio estimates and Wald confidence intervals for key attributes such as registration fees, chances of success, and cryopreservation duration, revealing their significant impact on decision–making. **Table 9** in **Appendix 1** presents the association between predicted probabilities and observed responses, demonstrating moderate predictive accuracy with a concordance rate of 69.7% and a c–statistic of 0.729. **Table 10** in **Appendix 1** further illustrates the choice patterns across predicted probability thresholds, where participants with higher predicted probabilities (over 0.5) tended to select the first option, underscoring the influence of key predictive factors.

**Table 5** shows the results of a binary logistic regression analysis, with added factors and demographic variables. From the additional parameters, Factor_Risk, age and income were significant. Adding parameters to the model had little effect on the model’s C–Index (rose from 72.9% in previous model to 74.5% in this model).

**Table 5:** Logistic regression for choice with added factors and demographics.

Analysis of Maximum Likelihood Estimates						
Parameter	DF	Estimate	Standard Error	Wald Chi–Square	Pr > ChiSq	Standardized Estimate
Intercept	1	–0.0516	0.2401	0.0462	0.8298	
Diff_Risk of infertility	1	0.00500	0.00115	19.0574	<.0001	0.0815
Diff_Chances of success of the oocyte cryopreservation process	1	–0.0252	0.00144	304.4951	<.0001	–0.3138
Diff_Chance of initiating a pregnancy from frozen oocyte	1	–0.0397	0.00231	295.4201	<.0001	–0.3080
Diff_Option of oocyte cryopreservation for chosen period of time (Years)	1	–0.2147	0.0152	200.0145	<.0001	–0.2791
Diff_Initial registration fee to fertility laboratory and cryopreservation (One–time payment) _100	1	0.0127	0.000764	275.1336	<.0001	0.3032
Diff_Annual fee for cryopreservation and must be paid every year (storage)	1	0.000445	0.000593	0.5628	0.4531	0.0123
Factor_Risk	1	0.2051	0.0417	24.1812	<.0001	0.0818
Factor_Price	1	–0.00809	0.0264	0.0940	0.7591	–0.00512
WTPYearly_100	1	0.00322	0.00178	3.2826	0.0700	0.0324
Age	1	–0.00977	0.00424	5.3114	0.0212	–0.0419
Religious	1	0.1294	0.0720	3.2283	0.0724	0.0325
Traditional	1	–0.0835	0.0685	1.4892	0.2223	–0.0216
Education	1	0.0378	0.0242	2.4486	0.1176	0.0254
Income	1	–0.0836	0.0317	6.9599	0.0083	–0.0492

In **Table 5**, additional explanatory variables were added to the model: The Risk Factor and Price Factor from part A of the study, WTP yearly, age, religious, traditional, education and income.

The significant parameters were the risk factor, age and income, but these added parameters had little effect on the model, as can be seen by the small, standardized estimates (0.04 to 0.08) and the small addition to the C–index (from 72.9% in previous model to 74.5% in this model).

The findings indicate that participants prioritize factors such as improved chances of future successful pregnancies and reduced anxiety about age–related fertility decline. This suggests that oocyte cryopreservation holds significant perceived value for them, offering considerable benefits that enhance their reproductive autonomy and overall well–being.

### 5.3 Key findings summary

The study employs statistical methods, including binary logistic regression and Conjoint Analysis (CA), to examine the factors that influence women’s decisions regarding oocyte cryopreservation. The findings emphasize the importance of several critical factors that influence these decisions, such as financial considerations, reproductive outcomes, and policy implications.

#### 5.3.1 Primary factors influencing decision–making

The results suggest that the perceived likelihood of attaining a successful pregnancy and concerns regarding future infertility are the most impactful factors on the decision to undergo oocyte cryopreservation. Cryopreservation is more likely to be considered by women if they believe it will substantially increase their chances of conceiving in the future. This demonstrates that the decision is not exclusively influenced by current circumstances, but also by expectations of future fertility and reproductive autonomy.

#### 5.3.2 Economic influence on decision–making

The decision–making process is significantly influenced by financial factors. The results of the analysis indicate that women with higher incomes are more likely to choose oocyte cryopreservation. For many individuals, the expenses associated with the procedure, which encompass initial retrieval, storage, and future *in vitro* fertilization (IVF), may be prohibitive. This implies that financial constraints restrict access to this technology, rendering it a more viable alternative for individuals with higher economic resources.

#### 5.3.3 Significance of personal and social context

The significance of personal circumstances and social conditions is also underscored in the study. Women who perceive themselves as having a high risk of infertility, whether as a result of age or medical conditions, are more inclined to engage in oocyte cryopreservation. Furthermore, decisions can be influenced by societal factors, including the public’s perception of fertility preservation and the availability of supportive policies.

#### 5.3.4 Results of conjoined analysis

The Conjoint Analysis quantifies preferences by analyzing various aspects of oocyte cryopreservation, including the probability of successful pregnancy, hazards, and costs. The results indicate that women prioritize attributes associated with reproductive outcomes over immediate financial considerations. This suggests that the prospective long–term advantages of preserving fertility are perceived as outweighing the initial expenses.

#### 5.3.5 Results of binary logistic regression

The binary logistic regression analysis determines the factors that are most predictive of the decision to cryopreserve oocytes. Age, income, and perceived risk of infertility are all substantial predictors. The analysis offers a model for comprehending the mechanisms by which these variables interact to influence decision–making, providing insights into the groups that are most likely to contemplate oocyte cryopreservation.

## 6 Discussion

In recent years, there has been a significant increase in both medical and non–medical female fertility preservation methods. The present study focuses on social oocyte cryopreservation among women, performed due to personal, professional, or financial reasons and motivated by the desire to preserve reproductive capacity, which naturally decreases with age (12, 13, 21, 151–153).

While there is a considerable amount of literature on social oocyte freezing, empirical studies focusing on the detailed behavioral and economic factors influencing women’s decisions to undergo social oocyte cryopreservation are relatively limited. This study aims to fill this gap by exploring these aspects. This study may shed light on an area where research is needed.

Additionally, the current study is pioneering as it offers a thorough analysis of social oocyte cryopreservation, with a primary emphasis on planned behavioral aspects and the inclusion of relevant economic factors in the decision–making process.

The study examines the factors that influence the intentions and subsequent behaviors of oocyte cryopreservation. The expanding availability of oocyte cryopreservation presents a distinctive opportunity to examine the willingness and inclination of individuals to make use of this technology. The primary theoretical framework for this analysis is the Theory of Planned Behavior (TPB), which establishes a direct correlation between beliefs and behavior (55–57). TPB is a suitable paradigm for comprehending decisions related to fertility preservation, as it posits that intentions are strong predictors of actual behavior (56). Furthermore, this investigation incorporates an economic asserted preference approach with TPB to economically quantify preferences, utilizing CA, to identify the combinations of attributes that most significantly influence decision–making (150, 154, 155)

To effectively investigate the economic stated preference in the context of the decision–making process of women regarding oocyte cryopreservation, it is essential to conduct a more thorough examination of the economic framework. This entails an assessment of the specific attributes that influence these decisions and an assessment of their relative significance.

### 6.1 Understanding trade–offs in decision–making

The economic stated preference framework provides an essential perspective for analyzing the trade–offs women assess when contemplating oocyte cryopreservation. This technique enables women to evaluate several factors, including the probability of future pregnancy success, infertility risks, and the related financial implications. The findings of the present study demonstrate that women value long–term reproductive autonomy, including the potential for future parenthood, more than immediate financial limitations, highlighting a societal trend where reproductive timing increasingly aligns with career and personal aspirations (156, 157). This conclusion corroborates previous research, indicating that women perceive oocyte cryopreservation as a means of securing reproductive autonomy despite the substantial initial expenses (40).

The present study further emphasizes that women view cryopreservation as providing psychological reassurance and alleviating anxiety associated with fertility decline. The evidence indicates that women who opted for cryopreservation experienced enhanced control over their reproductive future, accompanied by a notable decrease in the pressure linked to biological aging (158). The findings affirm that reproductive autonomy is pivotal in these decisions, aligning with extensive feminist discourse on empowerment via reproductive choice (113, 157). Furthermore, the role of evidence–based counseling in improving decision–making cannot be overstated. It provides women with the necessary information and support, enhancing their confidence in making educated reproductive decisions, as the current research indicates.

### 6.2 Incorporating behavioral insights into economic models

Incorporating the Theory of Planned Behavior (TPB) into economic models provides a comprehensive framework for understanding women’s fertility preservation choices. The Theory of Planned Behavior elucidates those behavioral intentions, influenced by attitudes, subjective norms, and perceived behavioral control, significantly predict actual behavior (56). According to the present study, strong social support and a favorable attitude toward oocyte cryopreservation had a significant impact on women’s decisions. The role of social support in women’s fertility preservation decisions is crucial, and this paper’s findings highlight the need for understanding in this area. Women who recognized increased cultural acceptability or familial support were more inclined to prioritize egg freezing despite financial obstacles (158).

The present findings indicate that perceived behavioral control, especially when overcoming financial or logistical obstacles, was pivotal in decision–making. Notwithstanding these challenges, women who showed confidence in their capacity to manage the cryopreservation technique were more inclined to undertake the procedure. This underscores the importance of integrating economic factors with behavioral insights to comprehensively understand the complexity of reproductive decision–making. This integration is crucial in providing an enlightened and informed perspective on women’s fertility preservation decisions. Integrating the Theory of Planned Behavior into the economic model enhanced the comprehension of the interplay between financial, social, and psychological elements influencing women’s decisions on fertility preservation.

### 6.3 Assessing the impact of financial constraints

The study’s findings demonstrate that financial constraints significantly impede oocyte cryopreservation, thereby impacting reproductive equity. A significant percentage of women in the lower–income category considered the expenses of cryopreservation to be excessive, although acknowledging the long–term reproductive benefits (157, 159, 160). This financial barrier intensifies inequalities in access to fertility preservation technologies, highlighting the necessity for policies that promote equitable access (40). The statistics indicate a significant disparity between the desire to preserve fertility and the available financial resources, highlighting the necessity for governmental measures, such as subsidies or insurance coverage, to alleviate these obstacles.

The findings strongly support the establishment of financial assistance programs to enhance accessibility for a diverse demographic. By alleviating the economic barriers to cryopreservation, we can foster reproductive equity and autonomy, ensuring that fertility preservation options are within reach for all women, regardless of their financial circumstances. This potential impact of financial assistance programs offers hope for a more equitable future in reproductive healthcare.

### 6.4 Evaluating policy implications

The present research indicates significant interest among women in oocyte cryopreservation, with participants demonstrating robust support for enhancing accessibility to the treatment. However, the current financial barriers are hindering this. Therefore, it is crucial to consider public financing as a solution (161, 162). Currently, Israel’s national health insurance covers medically essential therapies but excludes elective fertility preservation, including social oocyte cryopreservation. Based on the findings, governments should contemplate including cryopreservation in public health benefits, empowering more women to make informed decisions and preserve their fertility by their reproductive objectives (162).

An essential revelation from the present research is that financial constraints constitute the principal obstacle preventing women from pursuing oocyte cryopreservation. International data indicates that subsidized fertility preservation enhances accessibility, especially for women who may postpone childbearing for personal or professional reasons but lack the financial resources to preserve their fertility at the ideal moment. Aligning policy with potential users’ economic interests and reproductive goals is crucial in democratizing access to fertility preservation services and guaranteeing reproductive equity (157).

### 6.5 Consequences for policy

The research underscores the advantages of integrating oocyte cryopreservation into public health financing, hence improving accessibility for a broader population, especially individuals encountering financial barriers. The findings indicate that, in the absence of financial support, cryopreservation is unattainable for several women, exacerbating existing inequalities in reproductive healthcare. Offering financial assistance via subsidies or insurance schemes might equalize access and synchronize healthcare policy with women’s reproductive objectives, as demonstrated by the experiences of women in the present research (76, 163).

This regulatory change is expected to diminish the future necessity for more intense and expensive reproductive treatments. Enhancing the accessibility of oocyte cryopreservation for a broader demographic enables healthcare institutions to address women’s reproductive needs and promote equitable access more effectively.

### 6.6 Psychological and emotional factors

The study revealed considerable psychological advantages associated with oocyte cryopreservation. Women who choose to freeze their oocytes reported an enhanced sense of control over their reproductive prospects and a significant decrease in anxiety associated with fertility loss. The perception of emotional alleviation was often identified as a primary incentive for undertaking the procedure (54). The present study findings highlight the necessity of considering the medical and economic dimensions of cryopreservation and the psychological and emotional benefits it offers.

The psychological effects of oocyte cryopreservation are not uniform. While some women may experience a sense of control and reduced anxiety, others may persist in feeling anxious over success rates and future reproductive outcomes. This variability underscores the necessity for individualized and thorough counseling to alleviate these apprehensions. The emotional advantages, however, are often evident, underscoring the procedure’s significance in promoting reproductive autonomy and mental health.

### 6.7 Improving decision support tools

The financial and reproductive implications of cryopreservation can be better understood by women by developing decision support tools that are based on the economic stated preference framework. The decision–making process can be made more transparent and informed by personalized financial modelling, which can demonstrate the impact of various choices on individual circumstances (164). and enable women to make more informed decisions.

Ensuring equitable access to SEF is of utmost importance, alongside counseling and ethical considerations. Widespread public discourse, including that of popular media and social networks, has been deeply affected by the utilization and availability of SEF. Financial considerations for EF are significant, for many people, the cost of EF is prohibitively high and prevents access (165–169) SEF is a costly procedure, with expenses ranging from $15,000 to $20,000 per cycle, and it is usually not covered by insurance, rendering it an out–of–pocket cost (170). While the debate over insurance coverage for assisted reproductive technologies is vital, coverage for oocyte cryopreservation is mainly chiefly limited to medical cases rather than elective or nonmedical reasons. Consequently, insurance considerations are frequently omitted from SEF–specific financial discussions. A considerable number of patients require financial assistance in the form of loans or financial aid in order to afford the medications, procedures, and storage of retrieved oocytes required for cryopreservation. The aforementioned expenses include only the first retrieval and fail to consider any subsequent costs associated with thawing the oocytes for *in vitro* fertilization.

Additionally, patients may be required to repeat the refrigeration and IVF procedure multiple times in order to achieve the desired number of children. Supplementary expenses beyond the initial charges may pertain to the utilization of donor sperm or the testing of partner sperm prior to embryo development. As a result, numerous individuals may be unable to afford SEF (171),.

The study underscores the necessity of addressing economic disparities in the availability of oocyte cryopreservation. Women from lower socioeconomic backgrounds encounter numerous obstacles, including financial constraints, inadequate information, and inadequate social support (172, 173).

The analysis of CA data in this study indicates that the likelihood of success in oocyte cryopreservation and the initiation of pregnancy from frozen oocytes is substantially more significant than the reduced risk of age–related fertility decline. The relative significance of these factors indicates that there should be greater emphasis on enhancing the success rates and outcomes of oocyte cryopreservation procedures, rather than solely concentrating on the perceived risk of infertility. The importance of prioritizing and effectively communicating success rates to patients is underscored by this insight, which is essential for healthcare providers and policymakers.

The results also indicate that the perceived probability or concern of future infertility, financial capacity, and the perceived likelihood of conceiving a healthy child through the use of cryopreserved oocytes were all significant determinants.

The perceived probability of conceiving a healthy child through the use of cryopreserved oocytes is a compelling outcome that demands further investigation. This perspective has the potential to encourage women to opt for cryopreservation at an earlier age, as they may perceive it as a proactive approach to increase the likelihood of having healthy children, rather than relying solely on natural conception. This trend has the potential to transform the traditional narrative surrounding oocyte cryopreservation, establishing it as a favored method for assuring reproductive and genetic health, rather than merely a backup for age–related fertility decline. This realization could have substantial implications for the manner in which fertility preservation is communicated and perceived, indicating a necessity for targeted education and counseling to address the advantages and disadvantages of this method (174, 175). Furthermore, the research underscores the significance of age as a factor, as women’s fertility naturally decreases with age. Consequently, some people choose cryopreservation as a preventive measure against age–related fertility decline (12, 151–153). This is consistent with the findings of Stevenson et al. (152), who discovered that the decision to pursue oocyte cryo–preservation is substantially influenced by knowledge and perceptions about fertility decline.

Similar patterns of motivation and concern among women contemplating oocyte cryopreservation for non–medical purposes are revealed when the present paper’s findings are compared to those of studies, including those conducted by Tan et al. (154) and Stoop et al. (155). In 2014, Tan et al. (154) reported that Singaporean female medical students predominantly contemplated social oocyte freezing due to concerns about age–related fertility decline and the absence of a partner. This finding is consistent with the results of the present study analysis. In the same vein, Stoop et al. (155) discovered that the dread of future infertility was a significant factor in the decision of women in Belgium to undergo social oocyte cryopreservation.

This study also highlights a paradoxical finding, that younger women who cryopreserve their oocytes are less likely to use them later, as they often conceive naturally despite having preserved their oocytes as a precaution. This trend is consistent with Seyhan et al. (151), who reported that many women viewed cryopreservation as a “backup plan” rather than a primary strategy for childbearing.

The findings have several implications for clinical practice, healthcare policy, and ethical considerations. Clinicians and counselors should recognize the critical role that success rate sensitivity plays in patient decision–making. Transparent communication about the success rates of different cryopreservation options and personalized counselling can enhance decision quality and patient satisfaction. This is supported by the study by Stevenson et al. (152), which emphasizes the importance of patient education and counselling in fertility preservation decisions.

For healthcare services and marketing, clinics offering oocyte cryopreservation should ethically and accurately present their success rates to influence patient choice favorably. The competitive advantages should be highlighted in an ethical and precise manner in order to enable patients to make informed decisions. Policymakers should also consider developing guidelines that mandate the transparent reporting of success rates and other performance metrics for fertility preservation options, as suggested by Stoop et al. (155).

The study also identifies a prevalent lack of knowledge and comprehension among potential users, which contributes to the underutilization of oocyte cryopreservation. These disparities must be addressed by implementing educational initiatives and public health policies that are specifically designed to help. Additionally, the broader implementation of fertility treatments is impeded by the financial investment, technical complexity, and psychological distress that are associated with them, which underpins the necessity of enhanced accessibility and supportive policies.

### 6.8 Summarizing findings for policy and public understanding

The study essentially demonstrates that women are predominantly motivated to preserve their fertility due to concerns about future reproductive outcomes. Although financial obstacles are substantial, the perceived advantages of having the option to conceive at a later age often outweigh the costs. Public policies providing financial assistance for oocyte cryopreservation are crucial. Subsidies, insurance coverage, or integration into national health programs might mitigate the cost impact, allowing a broader demography to contemplate this choice. Enacting such legislation adheres to the tenets of reproductive justice, guaranteeing that all women, irrespective of socioeconomic background, can make informed choices regarding their reproductive destinies. Reducing financial obstacles enables greater access for women to fertility preservation technology, promoting reproductive autonomy and facilitating different family planning options. Empowering more women to make informed decisions about their reproductive futures through public policies that provide financial support for oocyte cryopreservation could help ensure broader access to this technology.

## 7 Conclusions

The study’s findings indicate that respondents place a high value on oocyte cryopreservation and perceive it as an effective technique for improving reproductive autonomy, reducing anxiety associated with fertility decline, and enhancing their overall well–being by expanding reproductive options. The study contributed to the quantification of women’s preferences by demonstrating that attributes such as the probability of a successful pregnancy and future reproductive opportunities are highly valued. This was achieved through the use of Conjoint Analysis (CA).

The nuanced understanding of how variations in success rates affect patient choices highlighted in this study emphasizes the need for clear, ethical, and effective communication and practices in the field of fertility treatments. These insights should guide clinicians, healthcare providers, and policymakers in their efforts to support patients in making the most informed and beneficial decisions regarding their fertility options.

This paper presents highly novel and as yet unpublished data offering behavioral and economic insights into women’s perceptions of oocyte cryopreservation and provides valuable insights for development of female reproductive health policy.

For women who want children, fertility education is key. Natural conception or donor insemination is the preferred way, and couples who have decided to have children should start trying early. For those, though, where conventional family planning is not possible due to various reasons, the chance for motherhood should not be denied, and they should be encouraged to be proactive to prevent infertility (176). The aim should be to increase awareness among women of reproductive age regarding age–related fertility decline. Funding strategies could potentially be developed in the future to prevent age–related fertility decline as preventative medicine has been developed in so many other fields. Also, algorithms could be developed to individually assess fertility status and cultivate a pro–fertility mentality in a realistic context (177).

## 8 Study limitations

Representativeness and Sample Bias: The study’s sample was restricted to Jewish Israeli women aged 18–65 from significant urban centers, which may not be representative of the broader population or diverse cultural contexts. The generalizability of the findings to women from other backgrounds or countries may be impacted by this limitation.

Self–Reported Data: The study is predicated on self–reported data. Self–reported data are susceptible to biases, including recall bias, social desirability bias, and the respondents’ propensity to disclose intimate or sensitive information accurately.

Preliminary Information: Participants in this study were provided with rudimentary information regarding oocyte cryopreservation, explaining advantages, risks, and the diverse circumstances in which it is considered, in order to guarantee that respondents were adequately informed. Nevertheless, this could have influenced their responses by predisposing them to view oocyte cryopreservation more favorably.

Cross–Sectional Design: The research employs a cross–sectional design, which records data at a singular point in time. This design restricts the capacity to establish causality or observe changes in attitudes or behaviors over time, which are essential for comprehending decision–making processes related to fertility preservation.

Conjoint Analysis Constraints: Respondents are required to make hypothetical decisions between predetermined scenarios in order to elicit preferences through the use of Conjoint Analysis (CA). This may not accurately represent the intricacies of real–world decision–making and may oversimplify the factors that influence women’s decisions regarding oocyte cryopreservation.

## Data Availability

The raw data supporting the conclusions of this article will be made available by the authors, without undue reservation.

## Footnotes

1 A subjective norm refers to an individual’s perception of the psychological support or social pressure exerted by their close social circle to either perform or refrain from a specific behavior or to achieve a particular goal. This perception is "subjective" because it may not accurately reflect the actual views of others or accepted societal norms. A more favorable attitude and subjective norm towards the decision to cryopreserve oocytes increase the likelihood of an individual to pursue it.

2 Fragile X Syndrome (FXS) is a genetic disorder caused by a mutation on the X chromosome, leading to intellectual disability and various behavioral and developmental issues.

3 Bio-scrutiny" refers to the detailed examination and regulation of biological and genetic characteristics within a community to ensure certain desired traits, such as physical health, genetic lineage, or cultural heritage. In Jewish communities, this often involves genetic testing and counselling to prevent hereditary diseases common among Ashkenazi Jews, such as Tay-Sachs or Gaucher disease. It can also extend to genealogical verification to confirm Jewish ancestry, which can be important for religious and cultural reasons, such as marriage eligibility.

4 A cross-sectional study is a type of observational research design that analyzes data from a population, or a representative subset, at one specific point in time. This method is particularly useful for understanding the current status of a phenomenon or to identify associations between variables without inferring causal relationships. In practice, cross-sectional studies collect data across a wide range of subjects at a single moment, aiming to capture a comprehensive picture of a particular research question.

5 ΔV is the change in utility in moving from the first scenario to the second scenario.

6 Risk of infertility is the difference in the percentage of the chances of becoming infertile between one scenario and the second scenario.

7 Chances of success of the oocyte cryopreservation process is the difference in the percentage of the chances of success of the oocyte cryopreservation process between one scenario and the second scenario.

8 Chances of initiating a pregnancy from frozen oocyte is the difference in the Chance of initiating a pregnancy from frozen oocyte between one scenario and the second scenario.

9 Option of oocyte cry presentation for chosen period of time is the difference between the Option of oocyte cryopreservation for chosen period between one scenario and the second scenario.

10 Initial registration fee to fertility laboratory and cryopreservation is the difference in the Initial registration fee to fertility laboratory and cryopreservation between one scenario and the second scenario.

11 Annual fee for cryopreservation and must be paid every year is the difference in the Annual fee for cryopreservation and must be paid every year between one scenario and the second scenario.

12 Factor Risk is the first explanatory factor.

13 Factor Price is the second explanatory factor.

14
*WTP Yearly* WTP Yearly is the willingness to pay for oocyte cryopreservation.

15 age, religion, income and education are the sociodemographic variables

16 e is the error term due to differences among observations

17 u is the error term due to differences among respondents

18 Age was used as a continuous variable.

19 In Israel, religious observance is a demographic factor that is used widely as a way for people to define themselves regarding their religious beliefs and practices. This is relevant when dealing with matters of procreation, which are regulated and circumscribed by religious law and doctrine.

20 The term ‘Orthodox’ refers to Jews who follow the traditional Jewish religion, a branch that strictly adheres to traditional teachings and interpretations of the Torah but is involved in the modern world.

21 The term ‘Ultra-Orthodox’ refers to Jewish communities that are highly conservative and maintain a strict adherence to traditional Jewish law and customs, maintaining insularity from the modern world. These communities are also known by the term "Haredi Judaism".

22 The term ‘secular' is not strictly defined, and it can mean either "not religious" or "convinced atheists"

23 The term ‘traditional' covers a wide range of ideologies and levels of observance and is based on self-definition

24 Education was considered a categorical variable with four categories:

25 Elementary School - 1st grade – 6^th^ grade, age range 6-12.

26 Partial High School - 7th grade – 9th grade age range 13-15.

27 High School full education - 10th grade – 12th grade, age range 16-18.

28 Post High School - 12th grade – 14th grade, age range 19-21.

29 Partial Academic degree - refers to the status of having completed some but not all of the requirements needed to earn a degree from an educational institution, college, university.

30 Academic degree full education - college, university

31 Since the study was conducted in Israel, the monetary values were measured in New Israel Shekels. I converted into dollars according to the dollar exchange rate on 23.02.2021, whereby, 1 ILS = 0.3063USD. The resulting dollar values were rounded according to mathematical rules for rounding numbers

32 Marital status was considered a categorical variable with four categories
