# Supplementary material for: Balancing choice and socioeconomic realities: analyzing behavioral and economic factors in social oocyte cryopreservation decisions
Source: Front Endocrinol (Lausanne). 2024 Dec 20;15:1467213. doi: 10.3389/fendo.2024.1467213 (PMC11695191; doi:10.3389/fendo.2024.1467213)
Supplement: Supplementary file 1 [file DataSheet2.docx]

***Appendix 2***

Hello

Let me introduce myself : I am Dr. Limor Gonen.

Welcome to my research study on social egg freezing, an increasingly popular choice among women seeking to preserve their reproductive potential for future use. This questionnaire is designed to gather valuable insights into the factors that influence the decision to opt for oocyte cryopreservation for non-medical reasons, commonly known as social egg freezing.

Your participation in this survey will contribute to a better understanding of how various personal, social, and economic factors play a role in this important decision. The information collected will be used to inform healthcare policy, improve support services for women considering this option, and enhance the overall knowledge surrounding fertility preservation techniques.

Confidentiality Assurance:

Please be assured that all responses will be kept strictly confidential. No personally identifiable information will be disclosed at any stage of this study, and all data will be reported in aggregate form only.

Survey Structure:

The questionnaire is divided into several sections, each focusing on different aspects of social egg freezing, including personal motivations, perceived social norms, economic considerations, and awareness of the procedure's medical aspects. It should take approximately 10-15 minutes to complete.

Instructions:

Please answer each question honestly based on your personal views and experiences.

If you are unsure about a question, please choose the response that is closest to your opinion.

There is no right or wrong answer; we are interested in your honest opinions.

Thank you for taking the time to participate in this important study. Your input is invaluable and will greatly assist in advancing research in the area of reproductive health and fertility preservation.

The following questions are related to a woman's desire to preserve her oocytes (eggs) for future use in case of a serious illness or even after her death. Women who may consider freezing their eggs or embryos usually fall under the categories of soldiers who are at risk of death or physical injury during military service (regular or reserve), patients with serious illnesses (who are about to undergo chemotherapy treatments for cancer and taking drugs that may damage oocyte quality), or women working in dangerous professions (such as factories with dangerous chemical substances or environmental pollution, X-ray or nuclear reactor, or risk-related jobs like firefighters, policemen, and construction workers).

Every woman is born with a fixed and non-renewable number of eggs, estimated to be around 2-3 million. By the time a woman reaches 37 years old, there is a significant decrease in the quantity and quality of her eggs. By the age of 45, there are almost no regular eggs left in her body.

Egg freezing significantly increases the chances of getting pregnant at a later age and allows women to use their own eggs instead of having to look for an egg donation.

In Israel, there is a distinction between women who want to freeze their eggs for medical reasons and women who want to freeze their eggs to preserve fertility. Women with medical reasons may be cancer patients (before chemotherapy and/or radiation) or those who, along with their partners, have fertility problems. Women with non-medical reasons are women who are not yet married and wish to preserve their fertility or career women who do not want to get pregnant but want to maintain their fertility for future use. This distinction is important because it affects the legal framework under which egg freezing takes place in Israel.

When you answer the questionnaire, imagine that you are personally faced with the decision whether to freeze eggs.

**Part I**

Please indicate the level of importance you assign to each of the following factors when deciding to freeze eggs. Rank the importance on a scale of 1 to 5, where 1 indicates "not important" and 5 indicates "very important".

| Factors | very important |  | | | not important |
| --- | --- | --- | --- | --- | --- |
| Risk of infertility (%) | 5 | 4 | 3 | 2 | 1 |
| Chances of success of the oocyte cryopreservation process (%) | 5 | 4 | 3 | 2 | 1 |
| Chance of initiating a pregnancy from cryopreserved oocyte (%) | 5 | 4 | 3 | 2 | 1 |
| Option of oocyte cryopreservation for chosen period of time (Years) | 5 | 4 | 3 | 2 | 1 |
| Price of initial registration to fertility laboratory and cryopreservation: One-time payment ($) | 5 | 4 | 3 | 2 | 1 |
| Annual fee for cryopreservation and storage ($) (must be paid every year) | 5 | 4 | 3 | 2 | 1 |

**Part II**

Imagine that you are facing a decision of whether to freeze your eggs. In the following questions, you will be presented with two different options, A and B. Each option has its own characteristics that make it different from the other. You will be asked to choose which option is better for you, based on the following criteria:

- Risk of infertility (%)
- Chances of success of the oocyte cryopreservation process (%)
- Chance of initiating a pregnancy from cryopreserved oocyte (%)
- Option of oocyte cryopreservation for a chosen period of time (Years)
- Price of initial registration to fertility laboratory and cryopreservation: One-time payment ($)
- Annual fee for cryopreservation and storage ($) (must be paid every year)

Please carefully examine each option separately and mark the preferred option in your opinion for all 12 given cases.

| **Scenario 1** | **Option A** | **Option B** |
| --- | --- | --- |
| Risk of infertility (%) | Low (less than 20%) | High (over 80%) |
| Chances of success of the oocyte cryopreservation process (%) | 80% | 30% |
| Chance of initiating a pregnancy from cryopreserved oocyte (%) | 10% | 30% |
| Option of oocyte cryopreservation for chosen period of time (Years) | 5 Years | 10 Years |
| Price of initial registration to fertility laboratory and cryopreservation: One-time payment ($) | $1838 | $1838 |
| Annual fee for cryopreservation and storage ($) (must be paid every year) | $45 | $15 |
| Which option do you prefer? | Option A | Option B |

| **Scenario 2** | **Option A** | **Option B** |
| --- | --- | --- |
| Risk of infertility (%) | Low (less than 20%) | High (over 80%) |
| Chances of success of the oocyte cryopreservation process (%) | 80% | 85% |
| Chance of initiating a pregnancy from cryopreserved oocyte (%) | 10% | 25% |
| Option of oocyte cryopreservation for chosen period of time (Years) | 5 Years | 5 Years |
| Price of initial registration to fertility laboratory and cryopreservation: One-time payment ($) | $1838 | $1838 |
| Annual fee for cryopreservation and storage ($) (must be paid every year) | $45 | $15 |
| Which option do you prefer? | Option A | Option B |

| **Scenario 3** | **Option A** | **Option B** |
| --- | --- | --- |
| Risk of infertility (%) | Low (less than 20%) | High (over 80%) |
| Chances of success of the oocyte cryopreservation process (%) | 80% | 30% |
| Chance of initiating a pregnancy from cryopreserved oocyte (%) | 10% | 25% |
| Option of oocyte cryopreservation for chosen period of time (Years) | 5 Years | 5 Years |
| Price of initial registration to fertility laboratory and cryopreservation: One-time payment ($) | $1838 | $3063 |
| Annual fee for cryopreservation and storage ($) (must be paid every year) | $45 | $45 |
| Which option do you prefer? | Option A | Option B |

| **Scenario 4** | **Option A** | **Option B** |
| --- | --- | --- |
| Risk of infertility (%) | Low (less than 20%) | High (over 80%) |
| Chances of success of the oocyte cryopreservation process (%) | 80% | 85% |
| Chance of initiating a pregnancy from cryopreserved oocyte (%) | 10% | 10% |
| Option of oocyte cryopreservation for chosen period of time (Years) | 5 Years | 10 Years |
| Price of initial registration to fertility laboratory and cryopreservation: One-time payment ($) | $1838 | 3676$ |
| Annual fee for cryopreservation and storage ($) (must be paid every year) | $45 | $45 |
| Which option do you prefer? | Option A | Option B |

| **Scenario 5** | **Option A** | **Option B** |
| --- | --- | --- |
| Risk of infertility (%) | Low (less than 20%) | High (over 80%) |
| Chances of success of the oocyte cryopreservation process (%) | 80% | 80% |
| Chance of initiating a pregnancy from cryopreserved oocyte (%) | 10% | 30% |
| Option of oocyte cryopreservation for chosen period of time (Years) | 5 Years | 5 Years |
| Price of initial registration to fertility laboratory and cryopreservation: One-time payment ($) | $1838 | $3063 |
| Annual fee for cryopreservation and storage ($) (must be paid every year) | $45 | $15 |
| Which option do you prefer? | Option A | Option B |

| **Scenario 6** | **Option A** | **Option B** |
| --- | --- | --- |
| Risk of infertility (%) | Low (less than 20%) | Low (less than 20%) |
| Chances of success of the oocyte cryopreservation process (%) | 80% | 85% |
| Chance of initiating a pregnancy from cryopreserved oocyte (%) | 10% | 50% |
| Option of oocyte cryopreservation for chosen period of time (Years) | 5 Years | 10 Years |
| Price of initial registration to fertility laboratory and cryopreservation: One-time payment ($) | $1838 | $3063 |
| Annual fee for cryopreservation and storage ($) (must be paid every year) | $45 | $15 |
| Which option do you prefer? | Option A | Option B |
| **Scenario 7** | **Option A** | **Option B** |
| Risk of infertility (%) | Low (less than 20%) | Low (less than 20%) |
| Chances of success of the oocyte cryopreservation process (%) | 80% | 80% |
| Chance of initiating a pregnancy from cryopreserved oocyte (%) | 10% | 25% |
| Option of oocyte cryopreservation for chosen period of time (Years) | 5 Years | 10 Years |
| Price of initial registration to fertility laboratory and cryopreservation: One-time payment ($) | $1838 | 0 |
| Annual fee for cryopreservation and storage ($) (must be paid every year) | $45 | $45 |
| Which option do you prefer? | Option A | Option B |

| **Scenario 8** | **Option A** | **Option B** |
| --- | --- | --- |
| Risk of infertility (%) | Low (less than 20%) | High (over 80%) |
| Chances of success of the oocyte cryopreservation process (%) | 80% | 30% |
| Chance of initiating a pregnancy from cryopreserved oocyte (%) | 10% | 50% |
| Option of oocyte cryopreservation for chosen period of time (Years) | 5 Years | 5 Years |
| Price of initial registration to fertility laboratory and cryopreservation : One-time payment ($) | $1838 | 0 |
| Annual fee for cryopreservation and storage ($) (must be paid every year) | $45 | $45 |
| Which option do you prefer? | Option A | Option B |

| **Scenario 9** | **Option A** | **Option B** |
| --- | --- | --- |
| Risk of infertility (%) | Low (less than 20%) | Low (less than 20%) |
| Chances of success of the oocyte cryopreservation process (%) | 80% | 50% |
| Chance of initiating a pregnancy from cryopreserved oocyte (%) | 10% | 10% |
| Option of oocyte cryopreservation for chosen period of time (Years) | 5 Years | 5 Years |
| Price of initial registration to fertility laboratory and cryopreservation : One-time payment ($) | $1838 | $3063 |
| Annual fee for cryopreservation and storage ($) (must be paid every year) | $45 | $45 |
| Which option do you prefer? | Option A | Option B |

| **Scenario 10** | **Option A** | **Option B** |
| --- | --- | --- |
| Risk of infertility (%) | Low (less than 20%) | High (over 80%) |
| Chances of success of the oocyte cryopreservation process (%) | 80% | 60% |
| Chance of initiating a pregnancy from cryopreserved oocyte (%) | 10% | 10% |
| Option of oocyte cryopreservation for chosen period of time (Years) | 5 Years | 5 Years |
| Price of initial registration to fertility laboratory and cryopreservation: One-time payment ($) | $1838 | 0 |
| Annual fee for cryopreservation and storage ($) (must be paid every year) | $45 | $15 |
| Which option do you prefer? | Option A | Option B |

| **Scenario 11** | **Option A** | **Option B** |
| --- | --- | --- |
| Risk of infertility (%) | Low (less than 20%) | Low (less than 20%) |
| Chances of success of the oocyte cryopreservation process (%) | 80% | 85% |
| Chance of initiating a pregnancy from cryopreserved oocyte (%) | 10% | 5% |
| Option of oocyte cryopreservation for chosen period of time (Years) | 5 Years | 5 Years |
| Price of initial registration to fertility laboratory and cryopreservation: One-time payment ($) | $1838 | $919 |
| Annual fee for cryopreservation and storage ($) (must be paid every year) | $45 | $45 |
| Which option do you prefer | Option A | Option B |

| **Scenario 12** | **Option A** | **Option B** |
| --- | --- | --- |
| Risk of infertility (%) | Low (less than 20%) | High (over 80%) |
| Chances of success of the oocyte cryopreservation process (%) | 80% | 50% |
| Chance of initiating a pregnancy from cryopreserved oocyte (%) | 10% | 25% |
| Option of oocyte cryopreservation for chosen period of time (Years) | 5 Years | 5 Years |
| Price of initial registration to fertility laboratory and cryopreservation: One-time payment ($) | $1838 | $919 |
| Annual fee for cryopreservation and storage ($) (must be paid every year) | $45 | $15 |
| Which option do you prefer? | Option A | Option B |

**Part III**

Please prioritize the following components by ranking them in order of importance. Assign the number 1 to the most important feature, 2 to the second most important feature, 3 to the third most important feature, and so on.

| Components | Ranking |
| --- | --- |
| Risk of infertility (%) |  |
| Chances of success of the oocyte cryopreservation process (%) |  |
| Chance of initiating a pregnancy from cryopreserved oocyte (%) |  |
| Option of oocyte cryopreservation for chosen period of time (Years) |  |
| Price of initial registration to fertility laboratory and cryopreservation: One-time payment ($) |  |
| Annual fee for cryopreservation and storage ($) (must be paid every year) |  |

**Part V**

You are requested to provide your opinion regarding the following statements related to this topic. You should indicate your level of agreement with each statement on the following answer scale:

| -1- I don't agree at all | -2- | -3- | -4- | -5- Agree moderately | -6- | -7- | -8- | -9- | -10- Agree completely |
| --- | --- | --- | --- | --- | --- | --- | --- | --- | --- |

- It is recommended that every woman should freeze her eggs in a hospital fertility laboratory.
- Every woman should consider freezing her eggs in case she is diagnosed with cancer in the future.
- Women who undertake dangerous activities should freeze their eggs as a precautionary measure.
- Parents who have lost their daughter to an illness may choose to find a surrogate mother and a suitable man who can give birth to a child from their family's frozen eggs to have grandchildren.
- It is advisable for women to consider the possibility of freezing their eggs.
- If my daughter were to undertake a dangerous activity, I would advise her to freeze her eggs beforehand to be safe.
- Women should only consider freezing their eggs if they are diagnosed with cancer ~~in the future~~.
- Parents who have lost their daughter to cancer may choose to find a surrogate mother and a suitable man who can give birth to a child from their family's frozen eggs.
- Freezing eggs ensures genetic continuity of the woman's family in case she passes away due to cancer.
- Freezing eggs ensures the spiritual continuity of the woman's family in case she passes away due to a dangerous activity.
- Eggs should only be removed from a deceased woman if she had explicitly stated her wish for eggs to be taken from her for procreation after her death.

**Part VI**

What is the maximum amount, in dollars, that you would be willing to pay to a fertility laboratory for depositing eggs each year for professional medical storage?

- 0
- 30.63-91.89 $
- 92.19-183.78 $
- 184.08-275.67 $
- 275.67-367.56 $
- 367.86-459.45 $
- 459.75-612.6 $
- More than $612.6 - state the sum ___________

**Part VII**

We kindly request that you provide us with some personal information. Please note that this information is confidential and will be used solely for research purposes.

1. Age
2. Gender
3. Degree of Religious Observance:

- Orthodox - Follow the traditional Jewish religion
- Ultra-Orthodox - Highly conservative
- Secular - Not religiously observant
- Traditional - Observant of some fundamentals of religious tradition

1. Education:

- Elementary school
- Partial high school
- High school - full education
- Post-high school
- Partial academic degree
- Academic degree

1. Personal monthly income $:

- $1,225.2 or less
- $1,225.5–$2,144.1
- $2,144.4–$3,063
- $3,063.3–$3,981.9
- $3,982.2 or more

1. Family Status:

- Married
- Single
- Divorced
- Widowed
