# Supplementary material for: Balancing choice and socioeconomic realities: analyzing behavioral and economic factors in social oocyte cryopreservation decisions
Source: Front Endocrinol (Lausanne). 2024 Dec 20;15:1467213. doi: 10.3389/fendo.2024.1467213 (PMC11695191; doi:10.3389/fendo.2024.1467213)
Supplement: Supplementary file 2 [file DataSheet1.docx]

**Appendix 1**

**Table 1- Attributes and Levels Included in the CA Study**

| **Levels and definition** | **Attribute** | |
| --- | --- | --- |
| Low (less than 20%); High (over 80%)  30; 50; 60; 80; 85 | | Risk of infertility (%)  Chances of success of the oocyte cryopreservation process (%) |
| 5; 10; 25; 30; 50 | | Chance of initiating a pregnancy from cryopreserved oocyte (%) |
| 5; 10 | | Option of oocyte cryopreservation for chosen period of time (Years) |
| 0; 919; 1838; 3063; 3676 | | Price of initial registration to fertility laboratory and cryopreservation  One-time payment ($) |
| 15; 45 | | The annual fee for cryopreservation and storage ($) (must be paid every year) |

**Table 2 - An Example of One of the Pairwise Choices**

| **Case 1** | | **Option B** | **Option A** |
| --- | --- | --- | --- |
| Risk of infertility | High (more than 80%) | | Low (less than 20%) |
| Chances of success of the oocyte cryopreservation process | 80% | | 30% |
| Chance of initiating a pregnancy from frozen oocyte | 10% | | 30% |
| Option of oocyte cryopreservation for chosen period of time (years) | 5 years | | 10 years |
| Initial registration fee to fertility laboratory and cryopreservation (one-time payment) | $1838 | | $1838 |
| The annual fee for cryopreservation and must be paid every year (storage) | $45 | | $15 |
| Which option do you prefer? | Prefer option B | | Prefer option A |

| **Table 3 - Scenarios (Diff=Option B- Option A)** | | | | | |  |  |  |
| --- | --- | --- | --- | --- | --- | --- | --- | --- |
| **Scenarios** | **Diff_ Risk of infertility** | **Diff_ Chances of success of the oocyte cryopreservation process** | **Diff_ Chance of initiating a pregnancy from frozen oocyte** | **Diff_ Option of oocyte cryopreservation for chosen period of time (years)** | **Diff_ Initial registration fee to fertility laboratory and cryopreservation (one-time payment)** | | **Diff_ Annual fee for cryopreservation and must be paid every year (storage)** | |
| 1 | 0 | -50 | 20 | 5 | 0 | | -100 |  |
| 2 | 60 | 5 | 15 | 0 | 0 | | -100 |  |
| 3 | 60 | -50 | 15 | 0 | 4000 | | 0 |  |
| 4 | 60 | 5 | 0 | 5 | 6000 | | 0 |  |
| 5 | 60 | 0 | 20 | 0 | 4000 | | -100 |  |
| 6 | 0 | 5 | 40 | 5 | 4000 | | -100 |  |
| 7 | 0 | 0 | 15 | 5 | -6000 | | 0 |  |
| 8 | 60 | -50 | 40 | 0 | -6000 | | 0 |  |
| 9 | 0 | -30 | 0 | 0 | 4000 | | 0 |  |
| 10 | 60 | -20 | 0 | 0 | -6000 | | -100 |  |
| 11 | 0 | 5 | -5 | 0 | -3000 | | 0 |  |
| 12 | 60 | -30 | 15 | 0 | -3000 | | -100 |  |

**Table 4 - Descriptives of Subject Choice**

|  | **%Col** |
| --- | --- |
| All | 100.00 |
| Case 1 | 53.30 |
| Chose First Option |  |
| Chose Second Option | 46.70 |
| Case 2 | 52.17 |
| Chose First Option |  |
| Chose Second Option | 47.83 |
| Case 3 | 73.35 |
| Chose First Option |  |
| Chose Second Option | 26.65 |
| Case 4 | 69.57 |
| Chose First Option |  |
| Chose Second Option | 30.43 |
| Case 5 | 59.47 |
| Chose First Option |  |
| Chose Second Option | 40.53 |
| Case 6 | 26.93 |
| Chose First Option |  |
| Chose Second Option | 73.07 |
| Case 7 | 20.76 |
| Chose First Option |  |
| Chose Second Option | 79.24 |
| Case 8 | 59.33 |
| Chose First Option |  |
| Chose Second Option | 40.67 |
| Case 9 | 87.38 |
| Chose First Option |  |
| Chose Second Option | 12.62 |
| Case 10 | 68.02 |
| Chose First Option |  |
| Chose Second Option | 31.98 |
| Case 11 | 58.91 |
| Chose First Option |  |
| Chose Second Option | 41.09 |
| Case 12 | 60.45 |
| Chose First Option |  |
| Chose Second Option | 39.55 |

**Table 5 - Descriptive Data for Choice by Scenario Parameters**

|  | | **Choose** | |
| --- | --- | --- | --- |
|  |  | **Didn't Choose** | **Chose** |
| Risk of infertility |  |  |  |
|  | Mean | 42.15 | 32.85 |
|  | Std | 28.96 | 24.62 |
| Chances of success of the oocyte cryopreservation process |  |  |  |
|  | Mean | 68.31 | 74.19 |
|  | Std | 19.56 | 16.25 |
| Chance of initiating a pregnancy from frozen oocyte |  |  |  |
|  | Mean | 16.43 | 18.15 |
|  | Std | 11.52 | 13.03 |
| Option of oocyte cryopreservation for chosen period of time (years) |  |  |  |
|  | Mean | 5.58 | 6.09 |
|  | Std | 1.60 | 2.07 |
| Initial registration fee to fertility laboratory and cryopreservation (one-time payment) |  |  |  |
|  | Mean | 6225.66 | 5607.67 |
|  | Std | 3169.00 | 2930.64 |
| The annual fee for cryopreservation and must be paid every year (storage) |  |  |  |
|  | Mean | 124.19 | 125.81 |
|  | Std | 43.76 | 42.83 |

**Table 6 - Descriptive Data for Choice by Difference in Scenario Parameters**

|  | | **Chose First Option** | **Chose Second Option** |
| --- | --- | --- | --- |
| Diff_Risk of infertility | N | 3743 | 3037 |
|  | Mean | 40.12 | 28.69 |
|  | Std | 28.24 | 29.98 |
| Diff_Chances of success of the oocyte cryopreservation process | N | 3743 | 3037 |
|  | Mean | -21.17 | -12.98 |
|  | Std | 22.18 | 22.28 |
| Diff_Chance of initiating a pregnancy from frozen oocyte | N | 3743 | 3037 |
|  | Mean | 11.65 | 18.20 |
|  | Std | 13.40 | 14.02 |
| Diff_Option of oocyte cryopreservation for chosen period of time (years) | N | 3743 | 3037 |
|  | Mean | 1.04 | 2.44 |
|  | Std | 2.03 | 2.50 |
| Diff_Initial registration fee to fertility laboratory and cryopreservation (one-time payment) | N | 3743 | 3037 |
|  | Mean | 408.76 | -875.86 |
|  | Std | 4256.56 | 4330.55 |
| Diff_Annual fee for cryopreservation and must be paid every year (storage) | N | 3743 | 3037 |
|  | Mean | -46.75 | -54.00 |
|  | Std | 49.90 | 49.85 |

**Table 7 - Factor Analysis After Varimax Rotation**

| **Rotated Factor Pattern** | | | | |
| --- | --- | --- | --- | --- |
|  | | | **Factor1** | **Factor2** |
| Risk of infertility |  | | 0.85867 | 0.00556 |
| Chances of success of the oocyte cryopreservation process | |  | 0.82169 | 0.13585 |
| Chance of initiating a pregnancy from cryopreserved oocyte | |  | 0.90584 | 0.16597 |
| Option of oocyte cryopreservation for chosen period of time | |  | 0.76152 | 0.32897 |
| Price of initial registration to fertility laboratory and cryopreservation  one-time payment | |  | 0.11187 | 0.93706 |
| The annual fee for cryopreservation and storage (must be paid every year) | |  | 0.18178 | 0.91633 |

| **Table 8 - Odds Ratio Estimates and Wald Confidence Intervals** | | | | |  |
| --- | --- | --- | --- | --- | --- |
| **Effect** | **Unit** | **Odds Ratio** | **95% Confidence Limits** | | |
| Diff_Risk of infertility | 1.0000 | 1.005 | 1.003 | 1.008 | |
| Diff_Chances of success of the oocyte  cryopreservation process | 1.0000 | 0.976 | 0.973 | 0.978 | |
| Diff_Chance of initiating a pregnancy from frozen oocyte | 1.0000 | 0.962 | 0.958 | 0.966 | |
| Diff_Option of oocyte cryopreservation for chosen period of time (Years) | 1.0000 | 0.807 | 0.785 | 0.831 | |
| Diff_Initial registration fee to fertility laboratory and cryopreservation (One-time payment) _100 | 1.0000 | 1.013 | 1.011 | 1.014 | |
| Diff_Annual fee for cryopreservation and must be paid every year (storage) | 1.0000 | 1.001 | 0.999 | 1.002 | |

From Table 8 odds ratios, we can see that larger differences in Chances of success of the oocyte, Chance of initiating a pregnancy from frozen oocyte, and Option of oocyte cryopreservation for chosen period of time (Years) lead to a lower probability of choosing the baseline option (OR<1), whereas larger differences in Initial registration fee to fertility laboratory and cryopreservation lead to a higher probability of choosing the baseline option (OR<1). The effect of difference in Annual fee for cryopreservation is NS, as can be seen by the fact that the value of "1" is included in the confidence interval for its OR.

| **Table 9 - Association of Predicted Probabilities and Observed Responses** | | | |
| --- | --- | --- | --- |
| Percent Concordant | 69.7 | Somers' D | 0.459 |
| Percent Discordant | 23.8 | Gamma | 0.491 |
| Percent Tied | 6.5 | Tau-a | 0.227 |
| Pairs | 11367491 | c | 0.729 |

| **Table 10 - Table of Choice by pred** | | | |
| --- | --- | --- | --- |
| **Choice** | **pred (Estimated Probability)** | | |
| Frequency Percent Row Pct Col Pct | <=0.5 | >0.5 | Total |
| Chose First Option | 710 10.47 18.97 31.42 | 3033 44.73 81.03 67.10 | 3743 55.21 |
| Chose Second Option | 1550 22.86 51.04 68.58 | 1487 21.93 48.96 32.90 | 3037 44.79 |
| Total | 2260 33.33 | 4520 66.67 | 6780 100.00 |

Table 10 shows that the C-Index for this model (discriminatory ability) is 73%, which indicates a reasonably good match. If we use said model to define an individual as choosing the baseline option, the model predicts a probability of more than 50% for this and choosing the other option, as defined by the difference variables, otherwise; the model correctly identifies 81% of the participants who chose the baseline option and 51% of the participants who chose the other option.
